# Genome-Wide Association Study Identifies Novel Restless Legs Syndrome Susceptibility Loci on 2p14 and 16q12.1

**DOI:** 10.1371/journal.pgen.1002171

**Published:** 2011-07-14

**Authors:** Juliane Winkelmann, Darina Czamara, Barbara Schormair, Franziska Knauf, Eva C. Schulte, Claudia Trenkwalder, Yves Dauvilliers, Olli Polo, Birgit Högl, Klaus Berger, Andrea Fuhs, Nadine Gross, Karin Stiasny-Kolster, Wolfgang Oertel, Cornelius G. Bachmann, Walter Paulus, Lan Xiong, Jacques Montplaisir, Guy A. Rouleau, Ingo Fietze, Jana Vávrová, David Kemlink, Karel Sonka, Sona Nevsimalova, Siong-Chi Lin, Zbigniew Wszolek, Carles Vilariño-Güell, Matthew J. Farrer, Viola Gschliesser, Birgit Frauscher, Tina Falkenstetter, Werner Poewe, Richard P. Allen, Christopher J. Earley, William G. Ondo, Wei-Dong Le, Derek Spieler, Maria Kaffe, Alexander Zimprich, Johannes Kettunen, Markus Perola, Kaisa Silander, Isabelle Cournu-Rebeix, Marcella Francavilla, Claire Fontenille, Bertrand Fontaine, Pavel Vodicka, Holger Prokisch, Peter Lichtner, Paul Peppard, Juliette Faraco, Emmanuel Mignot, Christian Gieger, Thomas Illig, H.-Erich Wichmann, Bertram Müller-Myhsok, Thomas Meitinger

**Affiliations:** 1 Institute of Human Genetics, Technische Universität München, Munich, Germany; 2 Department of Neurology, Technische Universität München, Munich, Germany; 3 Institute of Human Genetics, Helmholtz Zentrum München – German Research Center for Environmental Health, Neuherberg, Germany; 4 Max Planck Institute of Psychiatry, Munich, Germany; 5 Paracelsus-Elena-Hospital, Kassel, Germany; 6 Unité du Sommeil, Service de Neurologie, Hôpital Gui-de-Chauliac, INSERM U1061, Montpellier, France; 7 Department of Pulmonary Medicine, Tampere University Hospital, Tampere, Finland; 8 Sleep Research Unit, University of Turku, Turku, Finland; 9 Department of Neurology, Innsbruck Medical University, Innsbruck, Austria; 10 Institute of Epidemiology and Social Medicine, University Münster, Münster, Germany; 11 Somnomar, Sleep Research Institute, Marburg, Germany; 12 Department of Neurology, Center of Nervous Diseases, Philipps University, Marburg, Germany; 13 Department of Clinical Neurophysiology, University of Göttingen, Göttingen, Germany; 14 Centre of Excellence in Neuromics, CHUM Research Centre and the Department of Medicine, University of Montreal, Montreal, Canada; 15 Laboratoire d'étude des maladies du cerveau, Centre de recherche du CHUM, Hôpital Notre-Dame, Université de Montréal, Montréal, Canada; 16 Centre d'étude du sommeil, Hôpital du Sacré-Coeur de Montréal, Montréal, Canada; 17 Charite – Universitätsmedizin Berlin Interdisciplinary Center of Sleep Medicine, Berlin, Germany; 18 Department of Neurology, 1st Faculty of Medicine, Charles University, Prague, Czech Republic; 19 Centre for Molecular Medicine and Therapeutics, University of British Columbia, Vancouver, Canada; 20 Department of Neurology, Johns Hopkins University, Baltimore, Maryland, United States of America; 21 Department of Neurology, Baylor College of Medicine, Houston, Texas, United States of America; 22 Department of Neurology, Medical University of Vienna, Vienna, Austria; 23 Institute for Molecular Medicine Finland (FIMM), University of Helsinki, Helsinki, Finland; 24 Department of Chronic Disease Prevention, National Institute for Health and Welfare, Helsinki, Finland; 25 INSERM, UMR_S975, Paris, France; 26 Centre de Recherche Institut du Cerveau et de la Moelle, CNRS 7225, Paris, France; 27 Fédération des maladies du système nerveux, Pitié – Salpêtrière Hospital, AP-HP, Paris, France; 28 Institute of Experimental Medicine, Czech Academy of Sciences, Prague, Czech Republic; 29 Department of Population Health Sciences, University of Wisconsin, Madison, Wisconsin, United States of America; 30 Center For Narcolepsy, Stanford University, Palo Alto, California, United States of America; 31 Institute of Genetic Epidemiology, Helmholtz Zentrum München – German Research Center for Environmental Health, Neuherberg, Germany; 32 Unit for Molecular Epidemiology, Helmholtz Zentrum München – German Research Center for Environmental Health, Neuherberg, Germany; 33 Institute of Epidemiology I, Helmholtz Zentrum München – German Research Center for Environmental Health, Neuherberg, Germany; 34 Institute of Medical Informatics, Biometry, and Epidemiology, Chair of Epidemiology, Ludwig-Maximilians-Universität, Munich, Germany; 35 Klinikum Grosshadern, Munich, Germany; University of Oxford, United Kingdom

## Abstract

Restless legs syndrome (RLS) is a sensorimotor disorder with an age-dependent prevalence of up to 10% in the general population above 65 years of age. Affected individuals suffer from uncomfortable sensations and an urge to move in the lower limbs that occurs mainly in resting situations during the evening or at night. Moving the legs or walking leads to an improvement of symptoms. Concomitantly, patients report sleep disturbances with consequences such as reduced daytime functioning. We conducted a genome-wide association study (GWA) for RLS in 922 cases and 1,526 controls (using 301,406 SNPs) followed by a replication of 76 candidate SNPs in 3,935 cases and 5,754 controls, all of European ancestry. Herein, we identified six RLS susceptibility loci of genome-wide significance, two of them novel: an intergenic region on chromosome 2p14 (rs6747972, P = 9.03 × 10^−11^, OR = 1.23) and a locus on 16q12.1 (rs3104767, P = 9.4 × 10^−19^, OR = 1.35) in a linkage disequilibrium block of 140 kb containing the 5′-end of *TOX3* and the adjacent non-coding RNA *BC034767*.

## Author Summary

Restless legs syndrome (RLS) is one of the most common neurological disorders. Patients with RLS suffer from an urge to move the legs and unpleasant sensations located mostly deep in the calf. Symptoms mainly occur in resting situations in the evening or at night. As a consequence, initiation and maintenance of sleep become defective. Here, we performed a genome-wide association study to identify common genetic variants increasing the risk for disease. The genome-wide phase included 922 cases and 1,526 controls, and candidate SNPs were replicated in 3,935 cases and 5,754 controls, all of European ancestry. We identified two new RLS–associated loci: an intergenic region on chromosome 2p14 and a locus on 16q12.1 in a linkage disequilibrium block containing the 5′-end of *TOX3* and the adjacent non-coding RNA *BC034767*. *TOX3* has been implicated in the development of breast cancer. The physiologic role of TOX3 and BC034767 in the central nervous system and a possible involvement of these two genes in RLS pathogenesis remain to be established.

## Introduction

Restless legs syndrome (RLS) is a common neurological disorder with a prevalence of up to 10 %, which increases with age [1]. Affected individuals suffer from an urge to move due to uncomfortable sensations in the lower limbs present in the evening or at night. The symptoms occur during rest and relaxation, with walking or moving the extremity leading to prompt relief. Consequently, initiation and maintenance of sleep become defective [1]. RLS has been associated with iron deficiency, and is pharmacologically responsive to dopaminergic substitution. Increased cardiovascular events, depression, and anxiety count among the known co-morbidities [1].

Genome-wide association studies (GWAs) identified genetic risk factors within *MEIS1*, *BTBD9*, *PTPRD*, and a locus encompassing *MAP2K5* and *SKOR1* [2]–[4]. To identify additional RLS susceptibility loci, we undertook an enlarged GWA in a German case-control population, followed by replication in independent case-control samples originating from Europe, the United States of America, and Canada. In doing so, we identified six RLS susceptibility loci with genome-wide significance in the joint analysis, two of them novel: an intergenic region on chromosome 2p14 and a locus on 16q12.1 in close proximity to *TOX3* and the adjacent non-coding RNA *BC034767*.

## Results/Discussion

We enlarged our previously reported [2], [4] GWA sample to 954 German RLS cases and 1,814 German population-based controls from the KORA-S3/F3 survey and genotyped them on Affymetrix 5.0 (cases) and 6.0 (controls) arrays. To correct for population stratification, as a first step, we performed a multidimensional scaling (MDS) analysis, leading to the exclusion of 18 controls as outliers. In a second step, we conducted a variance components analysis to identify any residual substructure in the remaining samples, resulting in an inflation factor λ of 1.025 (Figures S1 and S2). The first four axes of variation from the MDS analysis were included as covariates in the association analysis of the genome-wide stage and all P-values were corrected for the observed λ.

**Figure 1:**
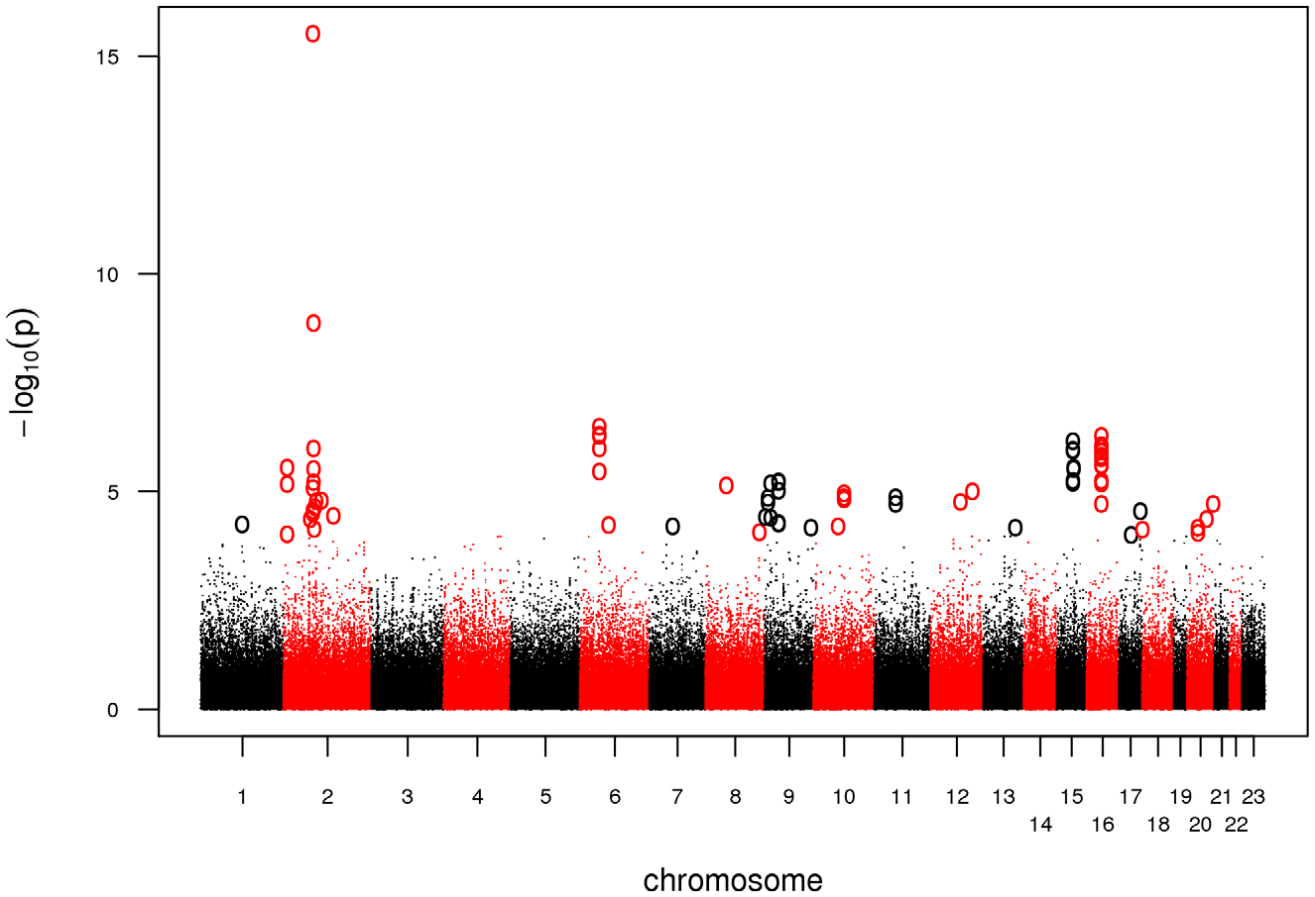
Manhattan plot of the GWA. Association results of the GWA stage. The x-axis represents genomic position along the 22 autosomes and the x-chromosome, the y-axis shows -log10(P) for each SNP assayed. SNPs with a nominal λ-corrected P<10^−4^ are highlighted as circles.

Prior to statistical analysis, genotyping data was subjected to extensive quality control. We excluded a total of 302 DNA samples due to a genotyping call rate <98 %. For individual SNP quality control, we adopted a stringent protocol in order to account for the complexity of an analysis combining 5.0 and 6.0 arrays. We excluded SNPs with a minor allele frequency (MAF) <5%, a callrate <98%, or a significant deviation from Hardy-Weinberg Equilibrium (HWE) in controls (P<0.00001). In addition, we dropped SNPs likely to be false-positive associations due to differential clustering between 5.0 and 6.0 arrays by adding a second set of cases of an unrelated phenotype and discarding SNPs showing association in this setup (see Materials and Methods). Finally, we tested 301,406 SNPs for association in 922 cases and 1,526 controls. Based on a threshold level of a nominal λ-corrected P_GWA_<10^-4^, a total of 47 SNPs distributed over 26 loci were selected for follow-up in the replication study (Figure 1, Table S1).

We genotyped these 47 SNPs together with 29 adjacent SNPs in strong linkage disequilibrium (LD, r^2^ = 0.5–0.9) using the Sequenom iPLEX platform in seven case-control populations of European descent, comprising a total of 3,935 cases and 5,754 controls. Eleven SNPs with a call rate <95%, MAF<5%, and P<0.00001 for deviation from HWE in controls as well as 432 samples with a genotyping call rate <90% were excluded. A set of 47 SNPs, genotyped in 186 samples on both platforms (Affymetrix and Sequenom), was used to calculate an average concordance rate of 99.24 %.

**Figure 2:**
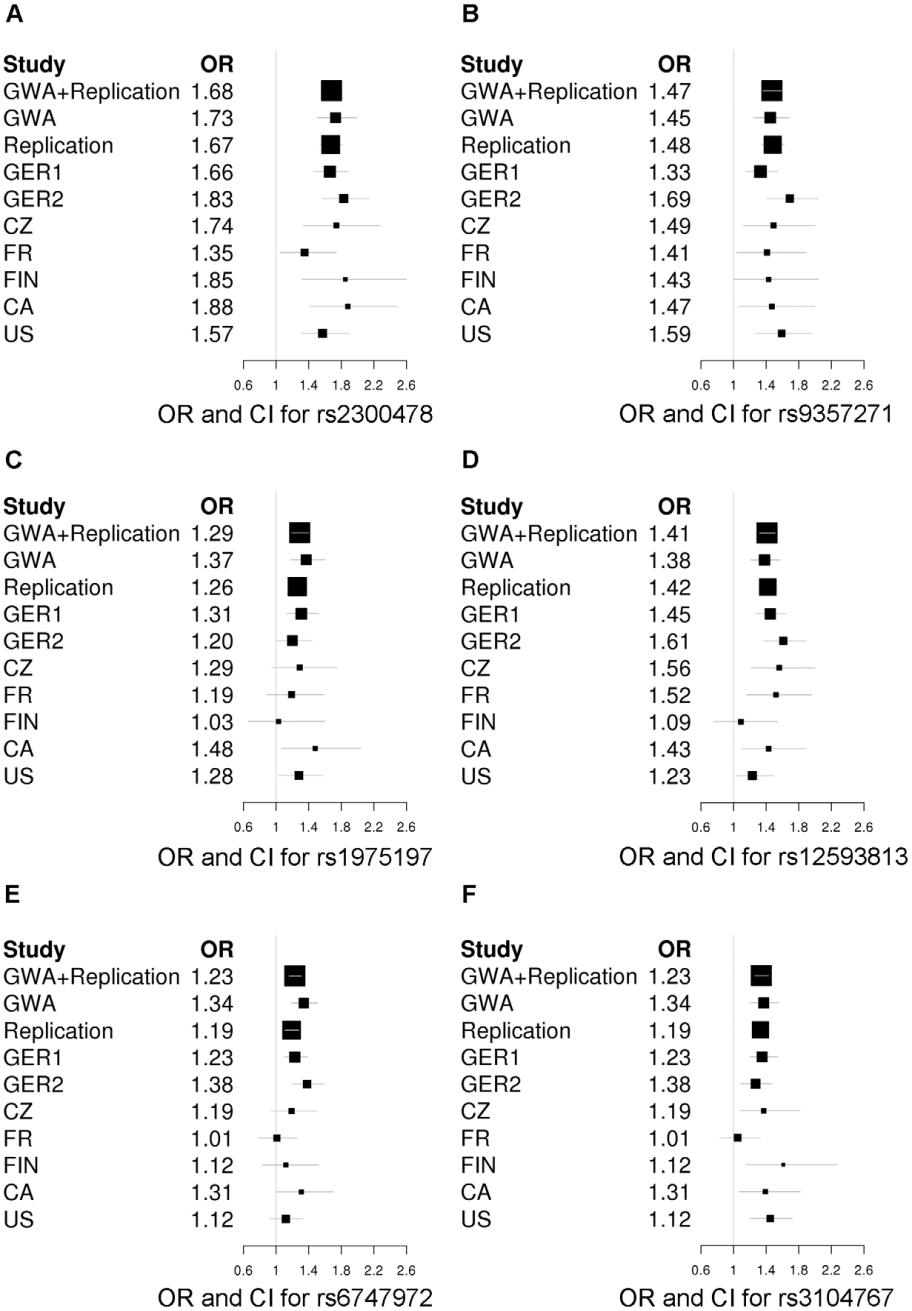
Forest plots of the RLS risk loci (1 SNP per locus). OR and corresponding confidence interval for the GWA sample, all individual replication samples, the combined replication sample as well as the combined GWA and replication sample are depicted. ORs are indicated by squares with the size of the square corresponding to the sample size for the individual populations. (A) rs2300478 in *MEIS1*; (B) rs9357271 in *BTBD9*; (C) rs1975197 in *PTPRD*; (D) rs12593813 in *MAP2K5/SKOR1*; (E) rs6747972 in intergenic region on chromosome 2; (F) rs3104767 in *TOX3/BC034767.*

The combined analysis of all replication samples confirmed the known four susceptibility loci and, in addition, identified two novel association signals on chromosomes 2p14 and 16q12.1 (Table 1). To address possible population stratification within the combined replication sample, we performed a fixed-effects meta-analysis. For four of the replication case-control populations, we included λ inflation factors which were available from a genomic controls experiment in a previous study in these populations [4]. These were used to correct the estimates for the standard error. Joint analysis of GWA and all replication samples showed genome-wide significance for these two novel loci as well as for the known RLS loci in *MEIS1, BTBD9*, *PTPRD*, and *MAP2K5/SKOR1* with a nominal λ -corrected P_JOINT_ <5×10^−8^ (Table 1). Depending on the variable power to detect the effects, the separate analyses of individual subsamples in the replication either confirmed the association after correction for multiple testing or yielded nominally significant results (Tables S2 and S3). The differing relevance of the risk loci in the individual samples is illustrated in forest plots (Figure 2). There was no evidence of epistasis between any of the six risk loci (P_Bonferroni_ >0.45).

**Table 1:** Association results of GWA and joint analysis of GWA and replication.

Chr	Locus	LD block (Mb)	SNP	Position (bp)	Risk allele	Risk allele frequency cases/controls	P_GWA_	P_REPLICATION_	P_JOINT_	Odds ratio (95% CI)
**Known risk loci (1 SNP per locus)**										
2	MEIS1	66.57–66.64	rs2300478	66634957	G	0.35/0.24	7.77×10^−16^	4.39×10^−35^	3.40×10^−49^	1.68 (1.57–1.81)
6	BTBD9	37.82–38.79	rs9357271	38473851	T	0.82/0.76	6.74×10^−7^	2.01×10^−16^	7.75×10^−22^	1.47 (1.35–1.47)
9	PTPRD	8.80–8.88	rs1975197	8836955	A	0.19/0.16	4.94×10^−5^	1.07×10^−6^	3.49×10^−10^	1.29 (1.19–1.40)
15	MAP2K5/SKOR1	65.25–65.94	rs12593813	65823906	G	0.75/0.68	1.49×10^−6^	1.54×10^−17^	1.37×10^−22^	1.41 (1.32–1.52)
**New genome-wide significant loci (P_JOINT_ < 5.2×10^−8^)**										
2	intergenic region	67.88–68.00	rs6747972	67923729	A	0.47/0.44	1.37×10^−6^	3.73×10^−6^	9.03×10^−11^	1.23 (1.16–1.31)
			rs2116050	67926267	G	0.49/0.47	7.84×10^−6^	4.85×10^−6^	4.83×10^−10^	1.22 (1.15–1.30)
16	TOX3/BC034767	51.07–51.21	rs3104767	51182239	G	0.65/0.58	7.38×10^−7^	2.16×10^−13^	9.40×10^−19^	1.35 (1.27–1.43)
			rs3104788	51196004	T	0.65/0.58	1.19×10^−6^	2.42×10^−13^	1.63×10^−18^	1.33 (1.25–1.43)

RLS-associated SNPs with genome-wide significance. P_GWA_, λ-corrected nominal P-value of GWA stage. P_REPLICATION_, nominal P-value obtained from meta-analysis of the replication stage samples. P_JOINT_, nominal P-value of the joint meta-analysis of GWA and replication stage, λ-corrected in samples where λ-values were available. Nominal P-values in GWA were calculated using logistic regression with sex, age, and the first four components from the MDS analysis of the IBS matrix as covariates. For nominal P_REPLICATION_ and P_JOINT_ -values, a fixed-effects inverse-variance meta-analysis was performed. Risk allele frequencies and odds ratios were calculated in the joint sample. LD blocks were defined by D' using Haploview 4.2 based on HapMap CEU population data from HapMap release #27. CI, 95% confidence interval. Genome positions refer to the Human March 2006 (hg18) assembly.

**Figure 3:**
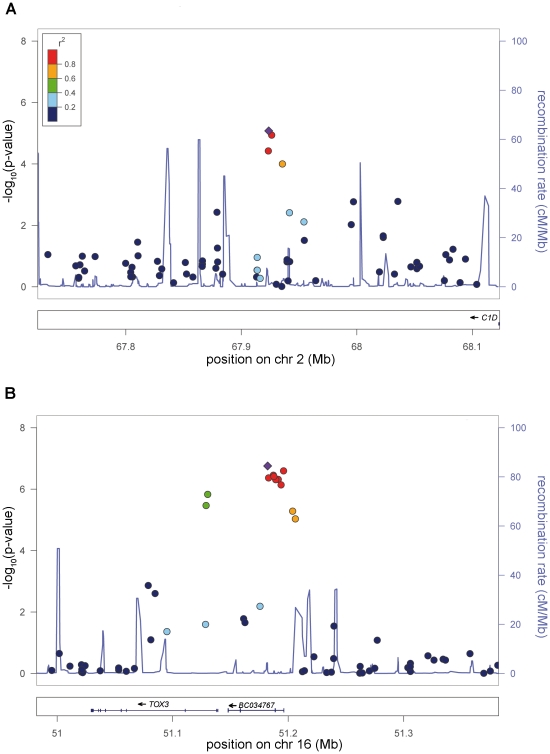
New genome-wide significant RLS loci. a) Risk locus on chromosome 2p14, showing the best-associated SNP rs6747972 and ±200 kb of surrounding sequence. b) Risk locus on chromosome 16p21, showing the best-associated SNP rs3104767 and ±200 kb of surrounding sequence. The left-hand x-axis shows the negative log10 of the nominal λ-corrected P-values of the GWA stage for all SNPs genotyped in the respective region. The right-hand x-axis shows the recombination frequency in cM/Mb. The y-axis shows the genomic position in Mb based on the hg18 assembly. The r^2^-based LD between SNPs is colour-coded, ranging from red (r^2^>0.8) to dark blue (r^2^<0.2) and uses the best-associated SNP as reference. This SNP is depicted as a violet diamond. Recombination frequency and r^2^ values are calculated from the HapMap II (release 22) CEU population. Plots were generated with LocusZoom 1.1 (http://csg.sph.umich.edu/locuszoom/).

The association signal on 2p14 (rs6747972: nominal λ-corrected P_JOINT_ = 9.03×10^−11^, odds ratio (OR)  = 1.23) is located in an LD block of 120 kb within an intergenic region 1.3 Mb downstream of *MEIS1* (Figure 3). Assuming a long-range regulatory function of the SNP-containing region, *in silico* analysis for clusters of highly conserved non-coding elements using the ANCORA browser (http://ancora.genereg.net) identified *MEIS1* as well as *ETAA1* as potential target genes [5], [6].

The second locus on chromosome 16q12.1 (rs3104767: nominal λ-corrected P_JOINT_  = 9.4×10^−19^, OR = 1.35) is located within an LD block of 140 kb (Figure 3), which contains the 5′UTR of *TOX3* (synonyms *TNRC9* and *CAGF9*) and the non-coding RNA *BC034767 (*synonym *LOC643714*). *TOX3* is a member of the high mobility box group family of non-histone chromatin proteins which interacts with *CREB* and *CBP* and plays a critical role in mediating calcium-dependent transcription in neurons [7]. GWAs have identified susceptibility variants for breast cancer in the identical region [8]. The best-associated breast cancer SNP, rs3803662, is in low LD (r^2^∼0.1, HapMap CEU data) with rs3104767, but showed association to RLS (λ-corrected nominal P_GWA_ = 7.29×10^−7^). However, logistic regression analysis conditioned on rs3104767 demonstrated that this association is dependent on rs3104767 (rs3803662: P_GWA/conditioned_ = 0.2883).

*BC034767* is represented in GenBank by two identical mRNA transcripts, BC034767 and BC029912. According to the gene model information of the UCSC and Ensembl genome browsers (http://genome.ucsc.edu and http://www.ensembl.org/index.html), these mRNAs are predicted to be non-coding. Additional *in silico* analysis using the Coding Potential Calculator (http://cpc.cbi.pku.edu.cn) supported this by attributing only a weak coding potential to this RNA, suggesting a regulatory function instead [9]. We also searched for rare alleles with strong effects and performed a mutation screening by sequencing all coding and non-coding exons of *TOX3* and *BC034767* in 188 German RLS cases (Table S4). In *TOX3*, a total of nine variants not listed in dbSNP (Build 130) were found, three of which are non-synonymous. Only one of these is also annotated in the 1000 Genomes project (November 2010 data release). Three additional new variants were located in putative exons 1 and 2 of *BC034767*. Analysis of the frequency of these variants as well as all known non-synonymous, frameshift, and splice-site coding SNPs in *TOX3* in a subset of one of the replication samples (726 cases and 735 controls from the GER1 sample) did not reveal any association to RLS. For a power of >80%, however, variants with an OR above 4.5 and a MAF ≥0.01 would be required. For even lower MAFs, ORs ≥10 would be necessary for sufficient power. Furthermore, the described CAG repeat within exon 7 of *TOX3* was not polymorphic as shown by fragment analysis in 100 population-based controls.

According to publicly available expression data (http://genome.ucsc.edu), in humans, *BC034767* is expressed in the testes only, while *TOX3* expression has been shown in the salivary glands, the trachea, and in the CNS. Detailed in-depth real time PCR profiling of *TOX3* showed high expression levels in the frontal and occipital cortex, the cerebellum, and the retina [10]. To assess a putative eQTL function of rs6747972 or rs3104767, we studied the SNP-genotype-dependent expression of *TOX3* and *BC034767* as well as of genes known to directly interact with *TOX3* (*CREB-1/CREBBP/CITED1*) and potential target genes of long-range regulatory elements at the locus on chromosome 2 (*MEIS1/ETAA1*) in RNA expression microarray data from peripheral blood in 323 general population controls [11]. No differential genotype-dependent expression variation was found.

To assess the potential for genetic risk prediction, we split our GWA sample in a training and a test set and determined classifiers for case-control status in the training set to predict case-control status in the test set. Training and test set were independent of each other – not only with respect to included individuals but also with respect to the genotyping procedure as we used genotypes generated on different genotyping platforms. As training set, we used those cases of the current GWA which had been genotyped on 500K arrays in a previous GWA and the corresponding control set [2], in total, 326 cases and 1,498 controls. The test set comprised 583 cases and 1,526 controls, genotyped on 5.0/6.0 arrays as part of the current study. Prior to the analysis, we removed the six known risk loci and performed LD-pruning to limit the analysis to SNPs not in LD with each other. In the end, a total of 76,532 SNPs were included in the pruned dataset. We conducted logistic regression with age and sex as covariates. Based on these association results, the sum score of SNPs showing the most significant effects (i.e. the number of risk alleles over all SNPs) weighted by the ln(OR) of these effects was chosen as predictor variable in the test set. We then varied the P-value threshold for SNPs included in the sum score. For a P-value <0.6, we observed a maximum area under the curve (AUC) of 63.9% and an explained genetic variance of 6.6% (Nagelkerke's R), values comparable to estimates obtained for other complex diseases such as breast cancer or diabetes (Table S5) [12]–[14]. Inclusion of the six known risk loci in this analysis resulted in a maximum AUC of 64.2% and an explained genetic variance of 6.8%.

Additionally, we performed risk prediction in the combined GWA and replication sample including only the six established RLS risk loci. For this purpose, we used the weighted risk allele score resulting in ORs of up to 8.6 (95% CI: 2.46–46.25) and an AUC of 65.1% (Figures S3 and S4).

By increasing the size of our discovery sample, we have identified two new RLS susceptibility loci. The top six loci show effect sizes between 1.22 and 1.77 and risk allele frequencies between 19 and 82%, and reveal genes in neuronal transcription pathways not previously suspected to be involved in the disorder.

## Materials and Methods

### Study population and phenotype assessment

#### Ethics statement

Written informed consent was obtained from each participant in the respective language. The study has been approved by the institutional review boards of the contributing authors. The primary review board was located in Munich, Bayerische Ärztekammer and Technische Universität München.

#### RLS patients (GWA and replication phase)

A total of 2,944 cases (GWA  = 954, replication  = 1,990) of European descent were recruited in two cycles via specialized outpatient clinics for RLS. German and Austrian cases for the GWA (GWA) and the replication sample (GER1) were recruited in Munich, Marburg, Kassel, Göttingen, Berlin (Germany, n in GWA = 830, n in GER1 = 1,028), Vienna, and Innsbruck (Austria, n in GWA = 124, n in GER1 = 288). The additional replication samples originated from Prag (Czech Republic (CZ), n = 351), Montpellier (France (FR), n = 182), and Turku (Finland (FIN), n = 141). In all patients, diagnosis was based upon the diagnostic criteria of the International RLS Study Group [1] as assessed in a personal interview conducted by an RLS expert. A positive family history was based on the report of at least one additional family member affected by RLS. We excluded patients with secondary RLS due to uremia, dialysis, or anemia due to iron deficiency. The presence of secondary RLS was determined by clinical interview, physical and neurological examination, blood chemistry, and nerve conduction studies whenever deemed clinically necessary.

In addition, 1,104 participants (GER2) of the “Course of RLS (COR-) Study”, a prospective cohort study on the natural course of disease in members of the German RLS patient organizations, were included as an additional replication sample. After providing informed consent, study participants sent their blood for DNA extraction to the Institute of Human Genetics, Munich, Germany. A limited validation of the RLS diagnosis among the majority of members was achieved through a diagnostic questionnaire. Five percent had also received a standardized physical examination and interview in one of the specialized RLS centers in Germany prior to recruitment. To avoid doublets, we checked these subjects against those recruited through other German RLS centers and excluded samples with identical birth date and sex.

556 cases (US) were recruited in the United States at Departments of Neurology at Universities in Baltimore, Miami, Houston, and Palo Alto. Diagnosis of RLS was made as mentioned above.

285 cases (CA) were recruited and diagnosed as above in Montréal, Canada. All subjects were exclusively of French-Canadian ancestry as defined by having four grandparents of French-Canadian origin.

Detailed demographic data of all samples are provided in Table S6.

#### Control populations (GWA and replication phase)

Controls for German and Austrian cases were of European descent and recruited from the KORA S3/F3 and S4 surveys, general population-based controls from southern Germany. KORA procedures and samples have been described [15]. For the GWA phase, we included 1,814 subjects from S3/F3, and, for the replication stage, 1,471 subjects from S4.

For replication of the GER2 sample, we used controls from the Dortmund Health Study (DHS), a population-based survey conducted in the city of Dortmund with the aim of determining the prevalence of chronic diseases and their risk factors in the general population. Sampling for the study was done randomly from the city's population register stratified by five-year age group and gender [16]. 597 subjects selected at random from the Czech blood and bone marrow donor registry served as Czech controls [17]. French controls included 768 parents of multiple sclerosis patients recruited from the French Group of Multiple Sclerosis Genetics Study (REFGENSEP) [18]. Finnish controls comprised 360 participants of the National FINRISK Study, a cross-sectional population survey on coronary risk factors collected every five years. The current study contains individuals recruited in 2002. Detailed description of the FINRISK cohorts can be found at www.nationalbiobanks.fi.

French-Canadian controls were 285 unrelated individuals recruited at the same hospital as the cases.

1,200 participants of the Wisconsin Sleep Cohort (WSC), an ongoing longitudinal study on the causes, consequences, and natural course of disease of sleep disorders, functioned as US controls [19].

None of the controls were phenotyped for RLS. All studies were approved by the institutional review boards in Germany, Austria, Czech Republic, France, Finland, the US, and Canada. Written informed consent was obtained from each participant. Detailed demographic data of all samples are provided in Table S6.

### Genotyping

#### GWA

Genotyping was performed on Affymetrix Genome-Wide Human SNP Arrays 5.0 (cases) and 6.0 (controls) following the manufacturer's protocol. The case sample included 628 cases from previous GWAs [2], [4] and 326 new cases. After genotype-calling using the BRLMM-P clustering algorithm [20], a total of 475,976 overlapping SNPs on both Affymetrix arrays were subjected to quality control. We added 655 cases of a different phenotype unrelated to RLS, genotyped on 5.0 arrays, to the analysis and excluded those SNPs which showed a significant difference of allele frequencies in cases (RLS and unrelated phenotype on 5.0) and controls (6.0) (n = 92). Thereby, we filtered out SNPs likely to be false-positive associations. We excluded SNPs with a minor allele frequency (MAF) <5% (n = 88,582), a callrate <98% (n = 65,906) or a significant deviation from Hardy-Weinberg Equilibrium (HWE) in controls (P<0.00001) (n = 20,060). Cluster plots of the GWA genotyping data for the best-associated SNPs in Table 1 are shown in Figure S5. Genotypes of these SNPs are available in Table S7.

#### Replication

We selected all SNPs with a λ-corrected P_nominal_<10^−4^ in the GWA for replication. These SNPs clustered in 26 loci (defined as the best associated SNP ±150 kb of flanking sequence). We genotyped a total of three SNPs in each of the 26 regions. These were either further associated neighbouring SNPs with a λ-corrected P_nominal_<10^−3^ or, in case of singleton SNPs, additional neighbouring SNPs from HapMap with the highest possible r^2^ (at least >0.5) with the best-associated SNP. We also genotyped the best-associated SNPs identified in the previous GWAs [2], [4].

Genotyping was performed on the MassARRAY system using MALDI-TOF mass spectrometry with the iPLEX Gold chemistry (Sequenom Inc, San Diego, CA, USA). Primers were designed using AssayDesign 3.1.2.2 with iPLEX Gold default parameters. Automated genotype calling was done with SpectroTYPER 3.4. Genotype clustering was visually checked by an experienced evaluator.

SNPs with a call rate<95%, MAF<5%, and P<0.00001 for deviations from HWE in controls were excluded. DNA samples with a call rate<90% were also excluded.

### Population stratification analysis

#### GWA

To identify and correct for population stratification, we performed an MDS analysis as implemented in PLINK 1.07 (http://pngu.mgh.harvard.edu/~purcell/plink, [21]) on the IBS matrix of our discovery sample. After excluding outliers by plotting the main axes of variation against each other, we performed logistic regression with age, sex, and the values of the MDS components as covariates. Using the Genomic Control approach [22], we obtained an inflation factor λ of 1.11.

Additionally, we performed a variance components analysis using the EMMAX software (http://genetics.cs.ucla.edu/emmax, [23]) and, again, calculated the inflation factor with Genomic Control, now resulting in a λ of 1.025. EMMAX uses a mixed linear model and does not only correct for population stratification but also for hidden relatedness. We, therefore, decided to base correction for population substructure on the EMMAX results.

#### Replication

Correction for population stratification was performed for the German, Czech, and the Canadian subsamples. The λ-values of 1.1032, 1.2286, and 1.2637 were derived from a previous Genomic Control experiment within the same samples using 176 intergenic or intronic SNPs [4]. Here, we had applied the expanded Genomic Control method GCF developed by Devlin and Roeder [24]. In the meta-analysis of all replication samples, the λ-corrected standard errors were included for the German, Czech, and Canadian samples. For the other replication samples from France, Finland, and the USA, no such data was available and, therefore, no correction factor was included in the analysis.

### Statistical analysis

Statistical analysis was performed using PLINK 1.07 (http://pngu.mgh.harvard.edu/~purcell/plink, [21]). In the GWA sample, we applied logistic regression with age, sex, and the first four axes of variation resulting from an MDS analysis as covariates.

P-values were λ-corrected with the λ of 1.025 from the EMMAX analysis. In the individual analysis of the single replication samples, we tested for association using logistic regression and correcting for gender and age as well as for population stratification where possible (see Population Stratification). Each replication sample was Bonferroni-corrected using the number of SNPs which passed quality control for the respective sample.

For the combined analysis of all replication samples, we performed a fixed-effects inverse-variance meta-analysis. Where available, we used λ-corrected standard errors in this analysis. Bonferroni-correction was performed for 74 SNPs, i.e. the number of SNPs which passed quality control in at least one replication sample.

For the joint analysis of the GWA and the replication samples, we also used a fixed-effects inverse-variance meta-analysis and again included λ-corrected values as far as possible. For the conditioned analysis, the SNP to be conditioned on was included as an additional covariate in the logistic regression analysis as implemented in PLINK.

Interaction analysis was performed using the –epistasis option in PLINK. Significance was determined via Bonferroni-correction (i.e. 0.05/28, as 28 SNP combinations were tested for interaction).

### Power calculation

Power calculation was performed using the CaTS power calculator [25] using a prevalence set of 0.08 and an additive genetic model (Table S3). The significance level was set at 0.05/74 for replication stage analysis and at 0.05/301,406 for genome-wide significance in the joint analysis of GWA and replication. For the rare variants association study, the significance level was set at 0.05/12.

### Mutation screening of *TOX3* and *BC034767*

All coding and non-coding exons including adjacent splice sites of *TOX3* (reference sequence NM_001146188) and *BC034767* (reference sequence IMAGE 5172237) were screened for mutations in 188 German RLS cases.

Mutation screening was performed with high resolution melting curve analysis using the LightScanner technology and standard protocols (IDAHO Technology Inc.). DNAs were analyzed in doublets. Samples with aberrant melting pattern were sequenced using BigDyeTerminator chemistry 3.1 (ABI) on an ABI 3730 sequencer. Sequence analysis was performed with the Staden package [26]. Primers were designed using ExonPrimer (http://ihg.gsf.de) or Primer3plus (www.bioinformatics.nl/cgi-bin/primer3plus/primer3plus.cgi). All identified variants were then genotyped in 735 RLS cases and 735 controls of the general population (KORA cohort) on the MassARRAY system, as described above.

In addition, fragment analysis of exon 7 of *TOX3* was performed to screen for polymorphic CAG trinucleotide repeats. DNA of 100 controls (50 females, 50 males) was pooled and analyzed on an ABI 3730 sequencer with LIZ-500 (ABI) as a standard. Primers were designed using Primer3plus, the forward Primer contains FAM for detection. Analysis was performed using GeneMapper v3.5.

### Expression analyses

Associations between *MEIS1/ETAA1* RNA expression and rs6747972 and between *TOX3/BC034767/CREB-1/CREBBP/CITED1* expression and rs3104767 were assessed using genome-wide SNP data (Affymetrix 6.0 chip) in conjunction with microarray data for human blood samples (n = 323 general population controls from the KORA cohort, Illumina Human WG6 v2 Expression BeadChip) [11]. A linear regression model conditioned on expression and controlling for age and sex was used to test for association.

### Prediction of genetic risk

#### Based on the performance of P-value-threshold selected SNPs in a training and a test sample

As training sample, we used those GWA-cases which had also been genotyped for our previous study [2]. We also included the control samples from this study. As a first quality control step, we carried out an association analysis comparing the Affymetrix 500K genotypes of these GWA-cases to the Affymetrix 5.0 genotypes of the same cases. Significant P-values would indicate systematic differences in the genotyping between the different chips. For further analysis, we only used those 259,302 SNPs with P-values >0.10. We performed a second quality control step in which IDs with a callrate below 98% and SNPs with a callrate below 98%, a MAF lower than 5%, or a P-value for deviation from HWE<0.00001 were removed.

Further, we excluded the four already known risk loci as well as the two newly identified loci and performed LD-pruning to limit the analysis to SNPs not in LD with each other. This was performed using a window-size of 50 SNPs. In each step, this window was shifted 5 SNPs. We used a threshold of 2 for the VIF (variance inflation factor). 76,532 SNPs, 326 cases, and 1,498 controls were included in the final training dataset. We conducted logistic regression with age and sex as covariates. Based on these association results, the sum score of SNPs showing the most significant effects (i.e. the number of risk alleles over all SNPs) weighted by the ln(OR) of these effects was chosen as predictor variable in the test set, comprising the remaining 583 cases of the GWA sample and 1,526 controls. None of these cases/controls were included in the training-sample, i.e. the test-sample constitutes a completely independent sample. Based on this sum score, we calculated the ROC curve and Nagelkerke's R to measure the explained variance.

#### Based on a weighted risk allele score

To evaluate the predictive value in our sample, we calculated a weighted sum score of risk alleles in the combined GWA and replication sample. To this end, we used one SNP from each RLS risk region and also included markers from the two newly identified regions on chromosome 16q12 and 2p14 (*MEIS1:* rs2300478, *2p14*: rs6747972, *BTBD9*: rs9296249, *PTPRD*: rs1975197, *MAP2K5:* rs11635424, *TOX3/BC034767*: rs3104767). At each SNP, the number of risk alleles was weighted with the corresponding ln(OR) for this SNP. The corresponding distribution of the score in cases and controls is illustrated in Figure S3. Employing this score for risk prediction resulted in an AUC of 0.651 (Figure S4).

## Supporting Information

Figure S1 MDS analysis plot for GWA. Distribution of cases (red) and controls (black) along the two main axes of variation identified in the MDS analysis. The three visible clouds are due to a common 3.8 Mb inversion polymorphism on chromosome 8 (described in: Tian C, Plenge RM, Ransom M, Lee A, Villoslada P, et al. (2008) Analysis and Application of European Genetic Substructure Using 300 K SNP Information. PLoS Genet 4: e4. doi:10.1371/journal.pgen.0040004).
(TIFF)

Figure S2 QQ-plot of GWA results. QQ-plot showing the P-value distribution before (red) and after (blue) correction for population stratification using Genomic Control.
(TIFF)

Figure S3 Weighted risk allele score analysis. Histogram of the weighted risk allele scores for cases and controls. The corresponding OR and CI for each category against the median category is depicted in green. The left y-axis refers to the number of individuals (in %), the right-axis refers to the OR values.
(TIFF)

Figure S4 ROC curve for weighted risk score analysis. Receiver operating characteristic (ROC) curve for the weighted risk allele score approach of risk prediction. The area under the curve (AUC) is 65.1%.
(TIFF)

Figure S5 Cluster plots of GWA genotyping for the six risk loci. For the best-associated SNPs at each risk locus, clusterplots were generated for cases and controls. Intensities of the A and B allele (based on the Affymetrix annotation of the SNPs) are given on the x- and y-axes and the respective genotypes are indicated in blue, green, and orange.
(PDF)

Table S1 GWA results for SNPs with λ-corrected P_GWA_<10–4 and additional SNPs selected for replication. A star (*) indicates SNPs which had been identified in previous RLS GWAs [2]–[4]. P-values of the GWA phase are given as λ-corrected nominal P-values. Two different methods for λ correction were applied, multi-dimensional-scaling (MDS)-analysis using PLINK and variance components (VC)-analysis using the EMMAX software with the P-values listed in the respective columns “MDS λ-corrected P_GWA_” and “VC λ-corrected P_GWA_”. The selection of SNPs for replication was based on the MDS λ-corrected P-values. r^2^-values based on Hapmap CEU data are given for those SNPs which were selected for replication based on their LD with the best-associated SNP in each region. Genomic position and gene annotation refer to the hg18 genome.
(DOC)

Table S2 Replication stage association results for individual replication samples. P-values are derived from logistic regression and correcting for gender and age as well as for population stratification where possible (see Materials and Methods). Each replication sample was Bonferroni-corrected using the number of SNPs which passed quality control for the respective sample. The OR refers to the minor allele. NA; SNP could not be analysed due to failing quality control in the respective sample.
(DOC)

Table S3 Power analysis for GWA, replication and joint analysis of GWA and replication. Power calculation was performed using the CaTS power calculator [25] using a prevalence set of 0.08 and an additive genetic model. The significance level α was set at 0.05/74 for replication stage analysis and at 0.05/301,406 for genome-wide significance in the joint analysis of GWA and replication.
(DOC)

Table S4 Results of TOX3 and BC034767 mutation screening. * “A” refers to the mutant allele, “B” to the reference allele. Position refers to hg18 genome annotation. Codon numbering refers to the reference sequence NM_001146188. Data of the 1000 genomes project was obtained from the November 2010 release via the 1000 genomes browser (http://browser.1000genomes.org/index.html).
(DOC)

Table S5 Prediction of genetic risk; training- and test-set approach. Inclusion threshold P-values were derived from a logistic regression with age and sex as covariates in the training sample. # SNPs indicates the number of SNPs passing the inclusion threshold. Based on these association results, the sum score of SNPs showing the most significant effects (i.e. the number of risk alleles over all SNPs) weighted by the ln(OR) of these effects was chosen as predictor variable in the test set. Based on this sum score, an AUC and Nagelkerke's R were calculated.
(DOC)

Table S6 Demographic data of GWA and replication samples. Mean age, mean age of onset and respective standard deviations and ranges are given in years. N: number of individuals; SD: standard deviation; AAO: age of onset. GWA: Genome-wide association study; CZ: Czechia; FR: France; FIN: Finland; CA: Canada; US: United States. - indicates that this information is not applicable for the respective sample.
(DOC)

Table S7 Genotype data of GWA samples. Genotypes of the GWA samples are given for the eight best-associated SNPs (see Table 1). SNP alleles are ACGT-coded. Phenotype information includes gender (1 =  male, 2 =  female) and disease status (1 =  unaffected, 2 =  affected).
(XLS)
