# Correction: Genome-Wide Association Study Identifies Novel Restless Legs Syndrome Susceptibility Loci on 2p14 and 16q12.1

**DOI:** 10.1371/annotation/393ad2d3-df4f-4770-87bc-00bfabf79362

**Published:** 2011-08-05

**Authors:** Juliane Winkelmann, Darina Czamara, Barbara Schormair, Franziska Knauf, Eva C. Schulte, Claudia Trenkwalder, Yves Dauvilliers, Olli Polo, Birgit Högl, Klaus Berger, Andrea Fuhs, Nadine Gross, Karin Stiasny-Kolster, Wolfgang Oertel, Cornelius G. Bachmann, Walter Paulus, Lan Xiong, Jacques Montplaisir, Guy A. Rouleau, Ingo Fietze, Jana Vávrová, David Kemlink, Karel Sonka, Sona Nevsimalova, Siong-Chi Lin, Zbigniew Wszolek, Carles Vilariño-Güell, Matthew J. Farrer, Viola Gschliesser, Birgit Frauscher, Tina Falkenstetter, Werner Poewe, Richard P. Allen, Christopher J. Earley, William G. Ondo, Wei-Dong Le, Derek Spieler, Maria Kaffe, Alexander Zimprich, Johannes Kettunen, Markus Perola, Kaisa Silander, Isabelle Cournu-Rebeix, Marcella Francavilla, Claire Fontenille, Bertrand Fontaine, Pavel Vodicka, Holger Prokisch, Peter Lichtner, Paul Peppard, Juliette Faraco, Emmanuel Mignot, Christian Gieger, Thomas Illig, H.-Erich Wichmann, Bertram Müller-Myhsok, Thomas Meitinger

An affiliation and its funding information were omitted from the published paper. Siong-Chi Lin and Zbigniew Wszolek are also affiliated with: Mayo Clinic, Jacksonville, Florida, United States of America. Research at the Mayo Clinic Florida was supported by NIH/NINDS P50NS072187 and Mayo Clinic Florida Research Committee CR program. 

