# Supplementary figures and images for: Genome-Wide Association Study Identifies Novel Restless Legs Syndrome Susceptibility Loci on 2p14 and 16q12.1
Source: PLoS Genet. 2011 Jul 14;7(7):e1002171. doi: 10.1371/journal.pgen.1002171 (PMC3136436; doi:10.1371/journal.pgen.1002171)

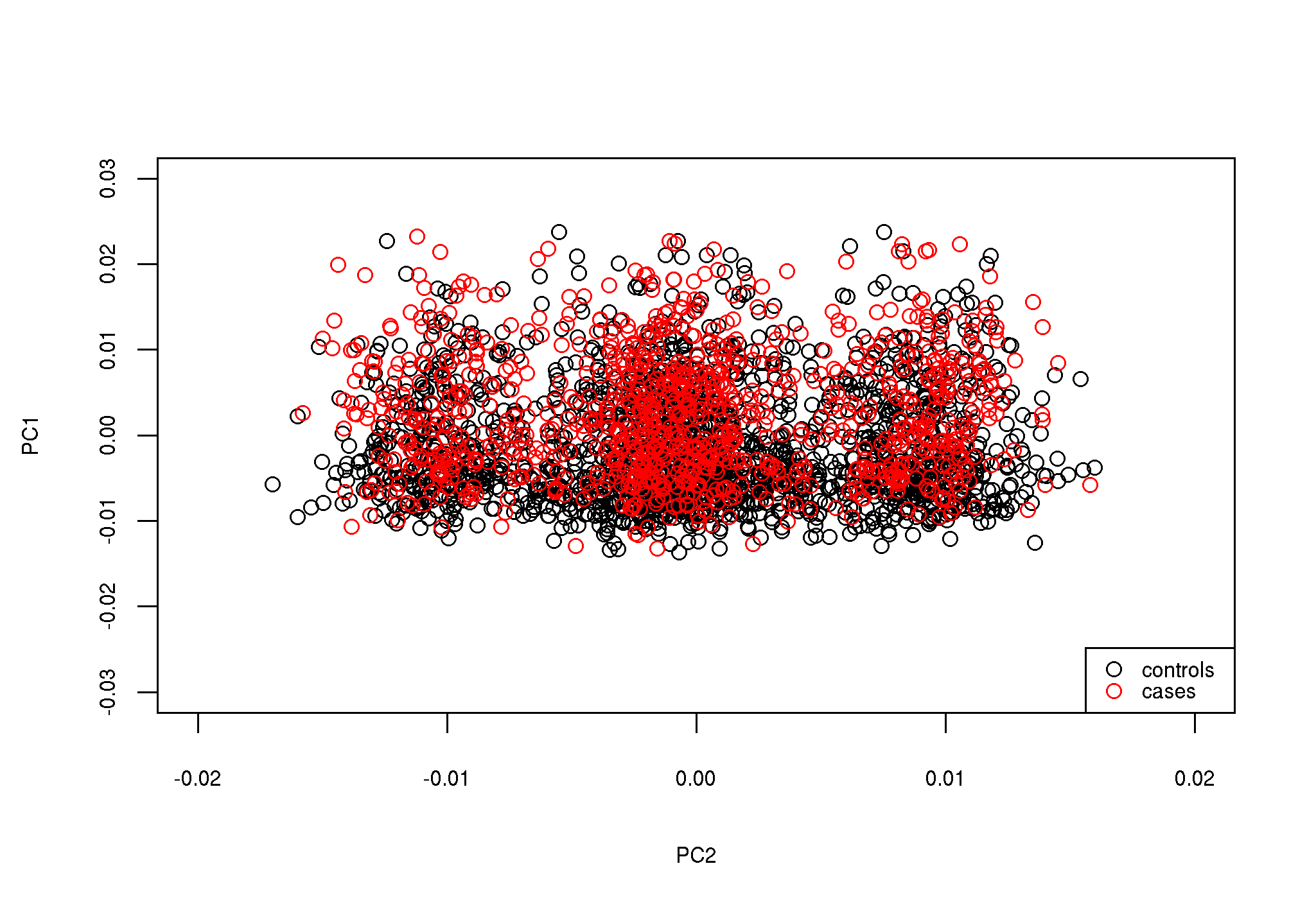

Supplement: Figure S1 — MDS analysis plot for GWA. Distribution of cases (red) and controls (black) along the two main axes of variation identified in the MDS analysis. The three visible clouds are due to a common 3.8 Mb inversion polymorphism on chromosome 8 (described in: Tian C, Plenge RM, Ransom M, Lee A, Villoslada P, et al. (2008) Analysis and Application of European Genetic Substructure Using 300 K SNP Information. PLoS Genet 4: e4. doi:10.1371/journal.pgen.0040004). (TIFF) [file pgen.1002171.s001.tif]

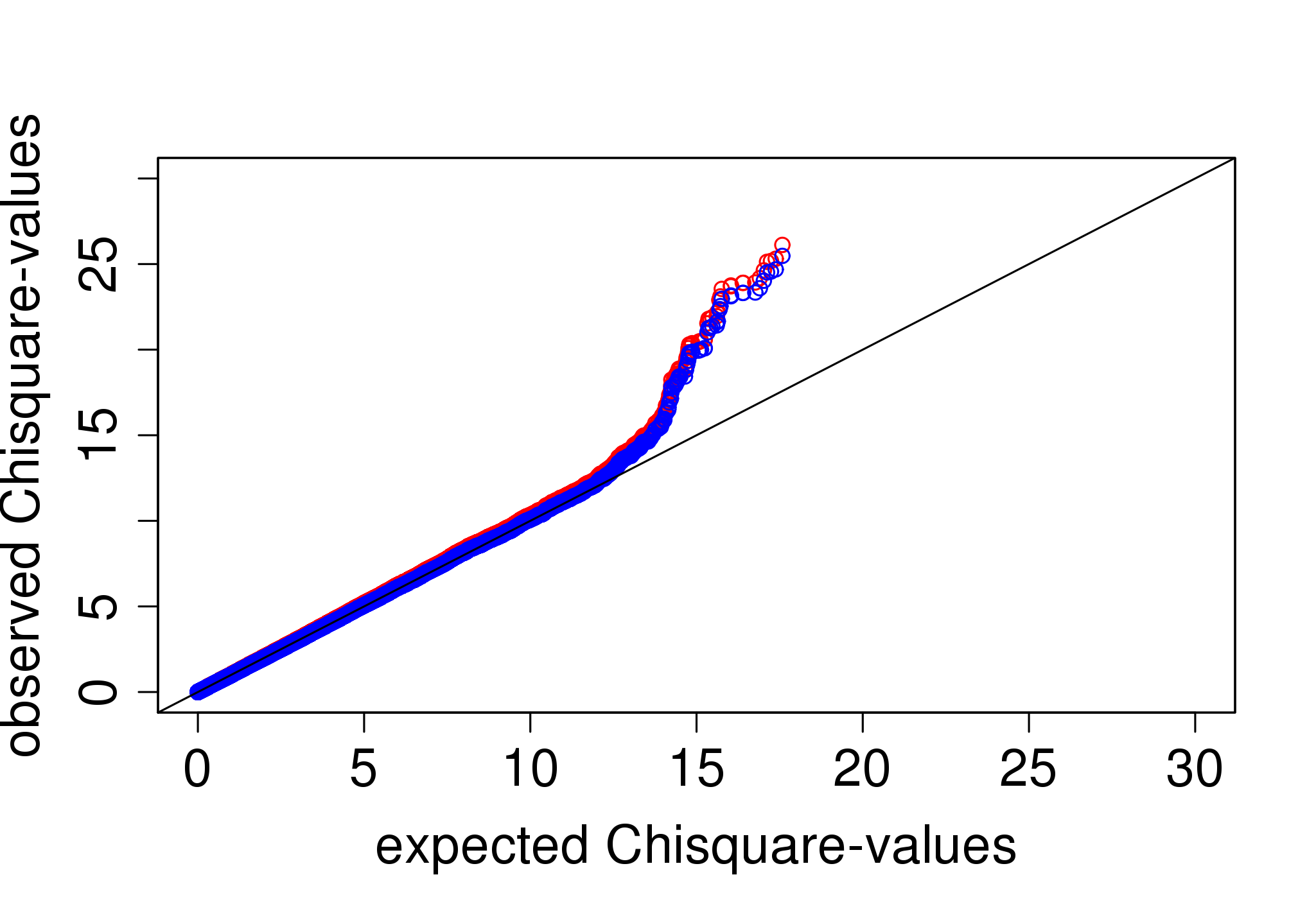

Supplement: Figure S2 — QQ-plot of GWA results. QQ-plot showing the P-value distribution before (red) and after (blue) correction for population stratification using Genomic Control. (TIFF) [file pgen.1002171.s002.tif]

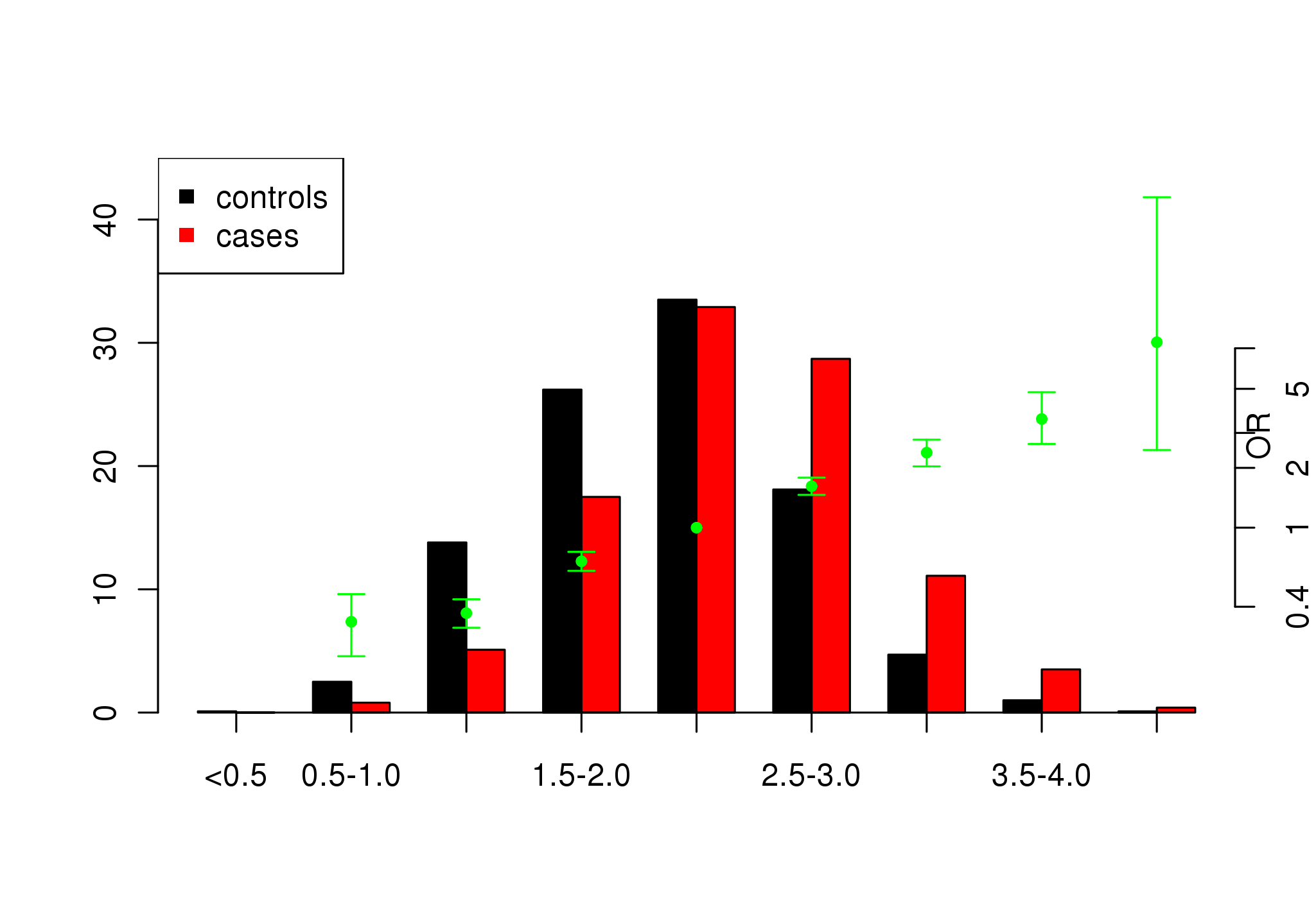

Supplement: Figure S3 — Weighted risk allele score analysis. Histogram of the weighted risk allele scores for cases and controls. The corresponding OR and CI for each category against the median category is depicted in green. The left y-axis refers to the number of individuals (in %), the right-axis refers to the OR values. (TIFF) [file pgen.1002171.s003.tif]

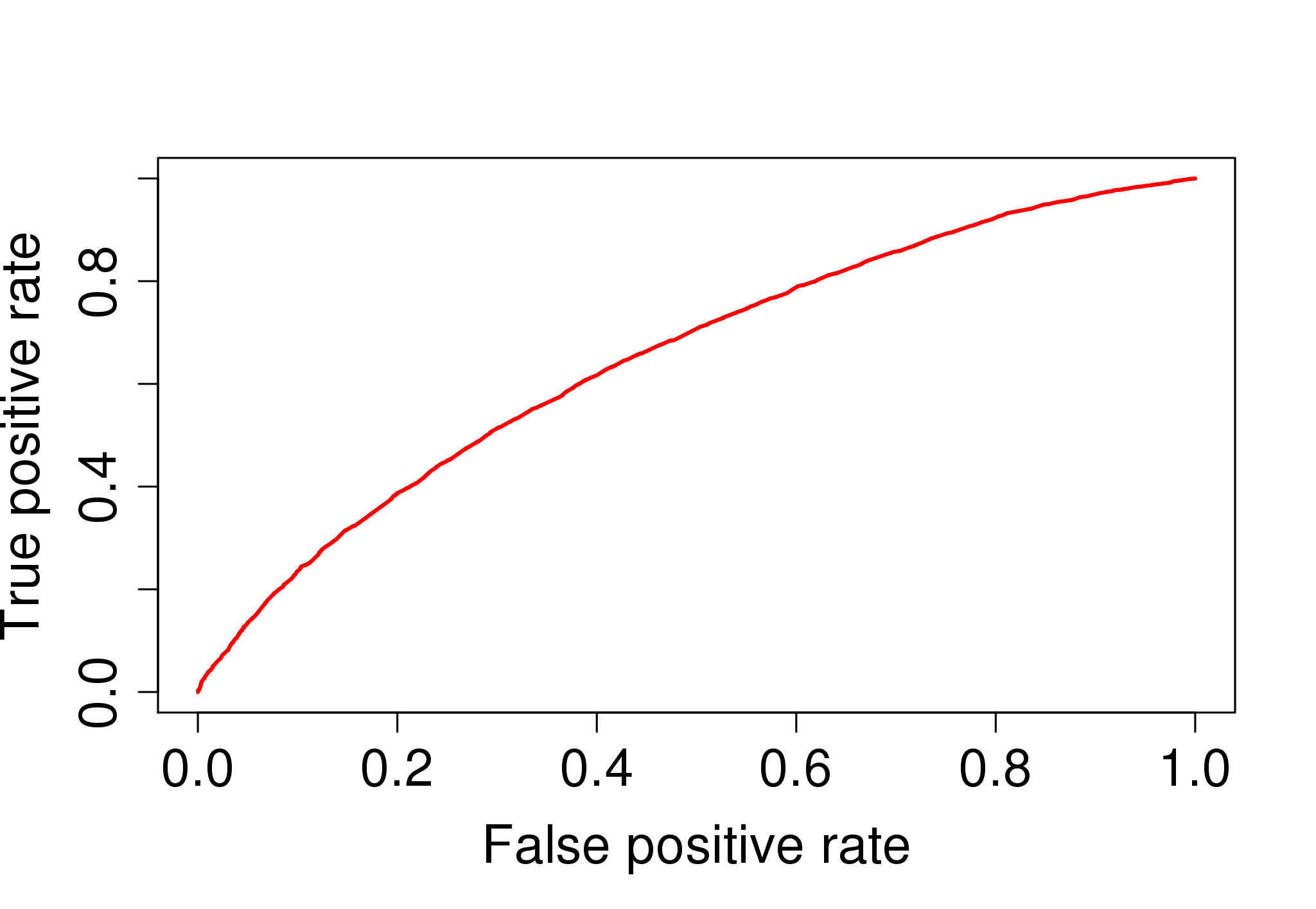

Supplement: Figure S4 — ROC curve for weighted risk score analysis. Receiver operating characteristic (ROC) curve for the weighted risk allele score approach of risk prediction. The area under the curve (AUC) is 65.1%. (TIFF) [file pgen.1002171.s004.tif]
