# Supplementary material for: Genome-Wide Association Study Identifies Novel Restless Legs Syndrome Susceptibility Loci on 2p14 and 16q12.1
Source: PLoS Genet. 2011 Jul 14;7(7):e1002171. doi: 10.1371/journal.pgen.1002171 (PMC3136436; doi:10.1371/journal.pgen.1002171)
Supplement: Figure S5 — Cluster plots of GWA genotyping for the six risk loci. For the best-associated SNPs at each risk locus, clusterplots were generated for cases and controls. Intensities of the A and B allele (based on the Affymetrix annotation of the SNPs) are given on the x- and y-axes and the respective genotypes are indicated in blue, green, and orange. (PDF) [file pgen.1002171.s005.pdf]

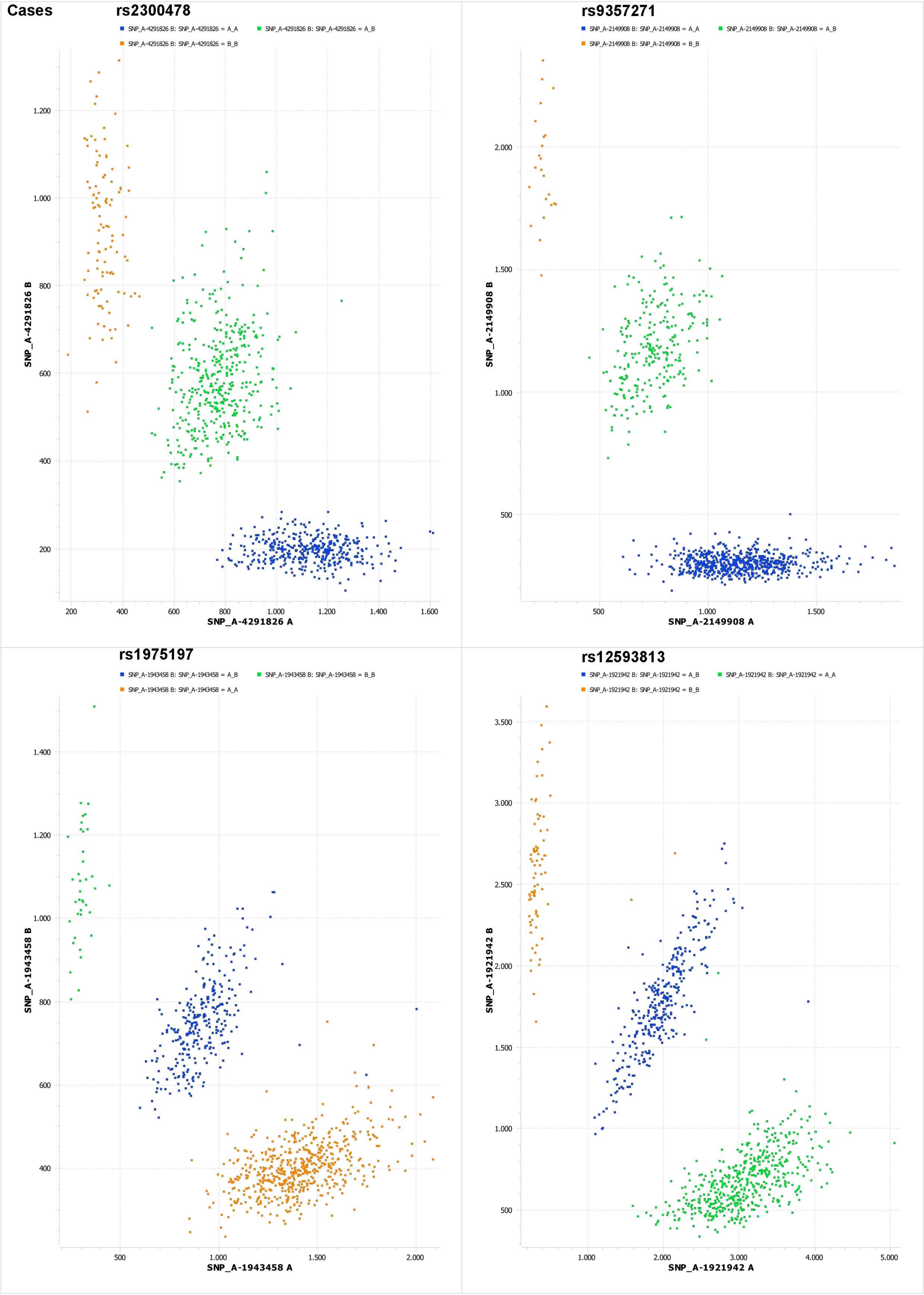
Cases
rs2300478
SNP_A-4291826 B: SNP_A-4291826 = A_A
SNP_A-4291826 B: SNP_A-4291826 = A_B
SNP_A-4291826 B: SNP_A-4291826 = B_B
SNP_A-4291826 B
SNP_A-4291826 A
rs9357271
SNP_A-2149908 B: SNP_A-2149908 = A_A
SNP_A-2149908 B: SNP_A-2149908 = A_B
SNP_A-2149908 B: SNP_A-2149908 = B_B
SNP_A-2149908 B
SNP_A-2149908 A
rs1975197
SNP_A-1943458 B: SNP_A-1943458 = A_B
SNP_A-1943458 B: SNP_A-1943458 = B_B
SNP_A-1943458 B: SNP_A-1943458 = A_A
SNP_A-1943458 B
SNP_A-1943458 A
rs12593813
SNP_A-1921942 B: SNP_A-1921942 = A_B
SNP_A-1921942 B: SNP_A-1921942 = A_A
SNP_A-1921942 B: SNP_A-1921942 = B_B
SNP_A-1921942 B
SNP_A-1921942 A

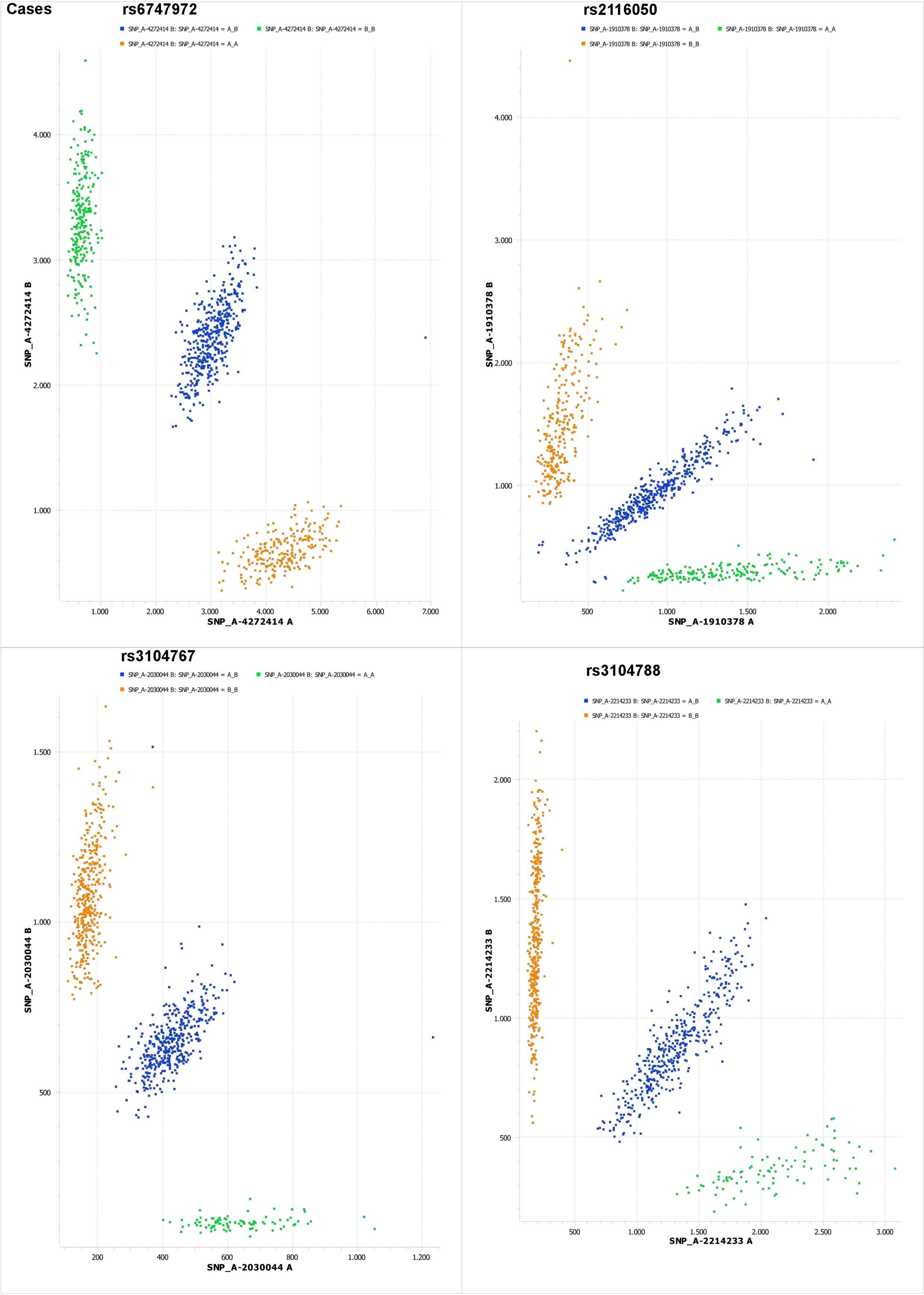
Cases
rs6747972
SNP_A-4272414 B: SNP_A-4272414 = A_B
SNP_A-4272414 B: SNP_A-4272414 = B_B
SNP_A-4272414 B: SNP_A-4272414 = A_A
SNP_A-4272414 B
SNP_A-4272414 A
rs2116050
SNP_A-1910378 B: SNP_A-1910378 = A_B
SNP_A-1910378 B: SNP_A-1910378 = A_A
SNP_A-1910378 B: SNP_A-1910378 = B_B
SNP_A-1910378 B
SNP_A-1910378 A
rs3104767
SNP_A-2030044 B: SNP_A-2030044 = A_B
SNP_A-2030044 B: SNP_A-2030044 = A_A
SNP_A-2030044 B: SNP_A-2030044 = B_B
SNP_A-2030044 B
SNP_A-2030044 A
rs3104788
SNP_A-2214233 B: SNP_A-2214233 = A_B
SNP_A-2214233 B: SNP_A-2214233 = A_A
SNP_A-2214233 B: SNP_A-2214233 = B_B
SNP_A-2214233 B
SNP_A-2214233 A

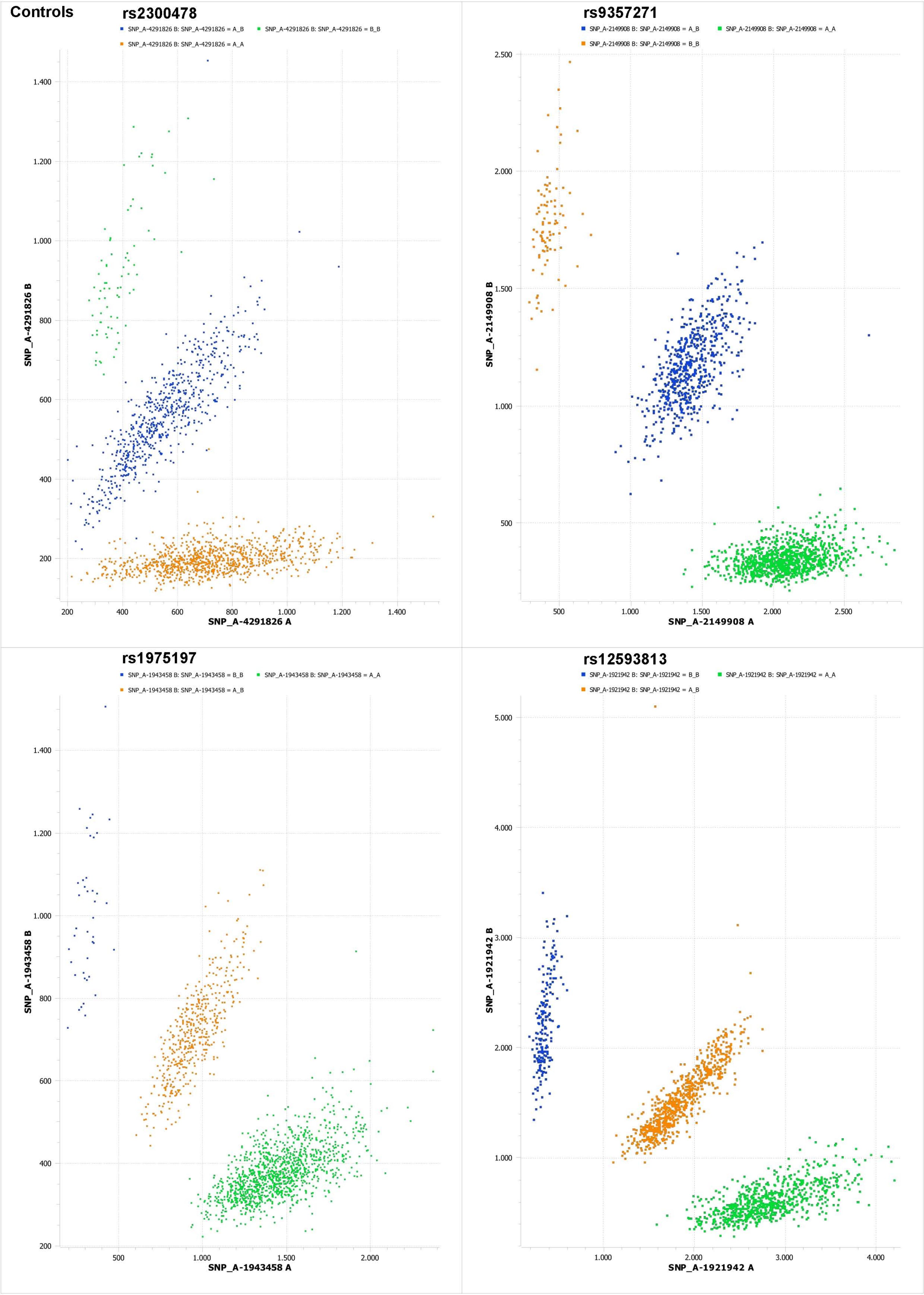
Controls
rs2300478
SNP_A-4291826 B: SNP_A-4291826 = A_B
SNP_A-4291826 B: SNP_A-4291826 = B_B
SNP_A-4291826 B: SNP_A-4291826 = A_A
SNP_A-4291826 B
SNP_A-4291826 A
rs9357271
SNP_A-2149908 B: SNP_A-2149908 = A_B
SNP_A-2149908 B: SNP_A-2149908 = A_A
SNP_A-2149908 B: SNP_A-2149908 = B_B
SNP_A-2149908 B
SNP_A-2149908 A
rs1975197
SNP_A-1943458 B: SNP_A-1943458 = B_B
SNP_A-1943458 B: SNP_A-1943458 = A_A
SNP_A-1943458 B: SNP_A-1943458 = A_B
SNP_A-1943458 B
SNP_A-1943458 A
rs12593813
SNP_A-1921942 B: SNP_A-1921942 = B_B
SNP_A-1921942 B: SNP_A-1921942 = A_A
SNP_A-1921942 B: SNP_A-1921942 = A_B
SNP_A-1921942 B
SNP_A-1921942 A

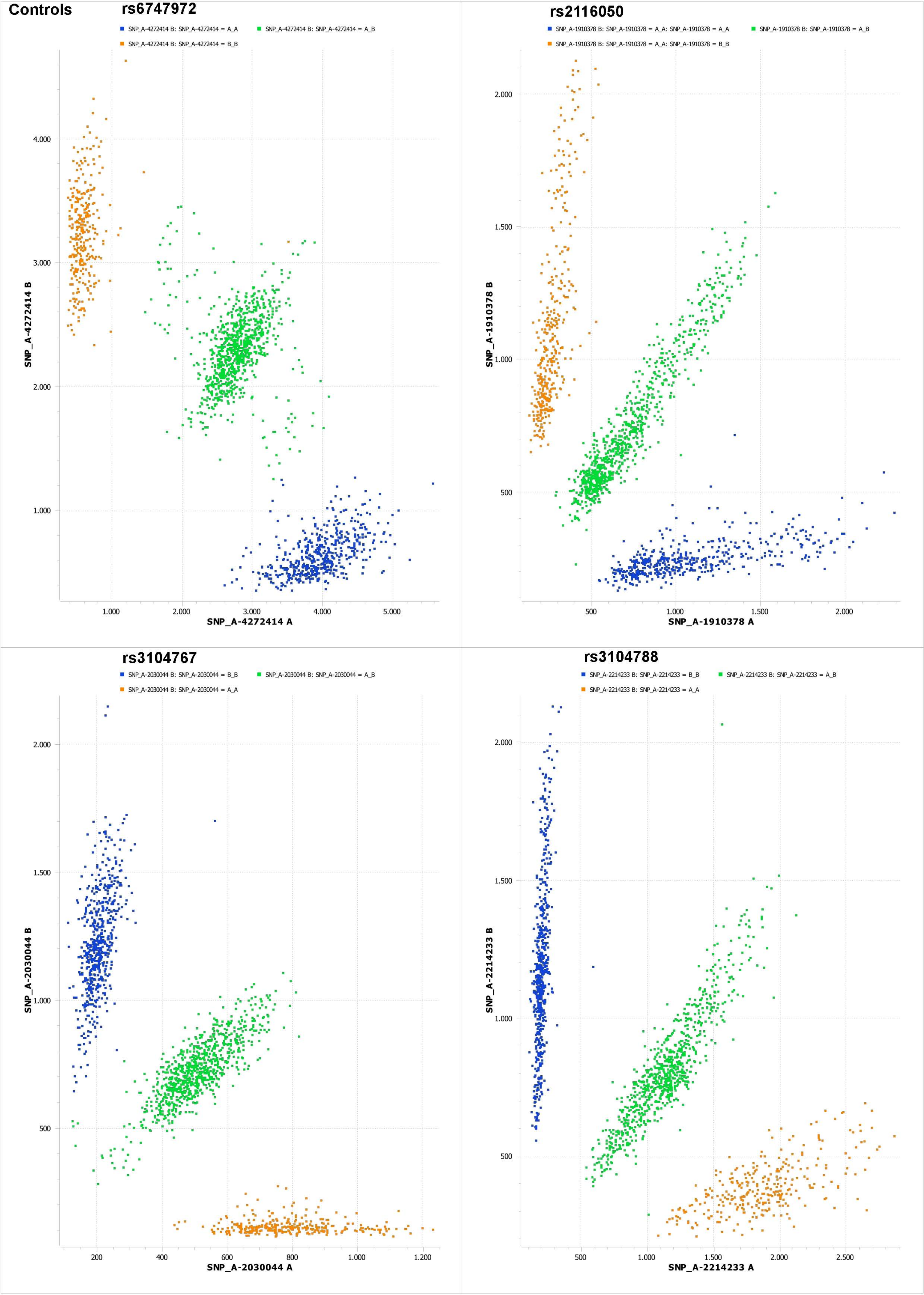
Controls
rs6747972
SNP_A-4272414 B: SNP_A-4272414 = A_A
SNP_A-4272414 B: SNP_A-4272414 = A_B
SNP_A-4272414 B: SNP_A-4272414 = B_B
SNP_A-4272414 B
SNP_A-4272414 A
rs2116050
SNP_A-1910378 B: SNP_A-1910378 = A_A: SNP_A-1910378 = A_A
SNP_A-1910378 B: SNP_A-1910378 = A_B
SNP_A-1910378 B: SNP_A-1910378 = A_A: SNP_A-1910378 = B_B
SNP_A-1910378 B
SNP_A-1910378 A
rs3104767
SNP_A-2030044 B: SNP_A-2030044 = B_B
SNP_A-2030044 B: SNP_A-2030044 = A_B
SNP_A-2030044 B: SNP_A-2030044 = A_A
SNP_A-2030044 B
SNP_A-2030044 A
rs3104788
SNP_A-2214233 B: SNP_A-2214233 = B_B
SNP_A-2214233 B: SNP_A-2214233 = A_B
SNP_A-2214233 B: SNP_A-2214233 = A_A
SNP_A-2214233 B
SNP_A-2214233 A
