# Supplementary material for: Genome-Wide Association Study Identifies Novel Restless Legs Syndrome Susceptibility Loci on 2p14 and 16q12.1
Source: PLoS Genet. 2011 Jul 14;7(7):e1002171. doi: 10.1371/journal.pgen.1002171 (PMC3136436; doi:10.1371/journal.pgen.1002171)
Supplement: Table S1 — GWA results for SNPs with λ-corrected PGWA<10–4 and additional SNPs selected for replication. A star (*) indicates SNPs which had been identified in previous RLS GWAs [2]–[4]. P-values of the GWA phase are given as λ-corrected nominal P-values. Two different methods for λ correction were applied, multi-dimensional-scaling (MDS)-analysis using PLINK and variance components (VC)-analysis using the EMMAX software with the P-values listed in the respective columns “MDS λ-corrected PGWA” and “VC λ-corrected PGWA”. The selection of SNPs for replication was based on the MDS λ-corrected P-values. r2-values based on Hapmap CEU data are given for those SNPs which were selected for replication based on their LD with the best-associated SNP in each region. Genomic position and gene annotation refer to the hg18 genome. (DOC) [file pgen.1002171.s006.doc]

### Table S 1: GWA results for SNPs with λ-corrected P_GWA_ < 10-4 and additional SNPs selected for replication.

| **Locus** | **Chr** | **Genomic position** | **SNP rs ID** | **Gene** | **MDS**  **λ-corrected P_GWA_** | **VC**  **λ-corrected P_GWA_** | **r^2^** |
| --- | --- | --- | --- | --- | --- | --- | --- |
| 1 | 1 | 63,644,346 | rs2273122 | ALG6 | 7.68E-05 | 8.72E-004 |  |
|  |  | 63,755,548 | rs12078371 | ITGB3BP | 1.29E-05 | 2.04E-004 |  |
|  |  | 63,849,402 | rs7528129 | PGM1 | 2.35E-05 | 2.17E-004 |  |
| 2 | 1 | 115,644,333 | rs4076018 | NGF | 7.63E-05 | 7.17E-005 |  |
|  |  | 115,647,744 | rs12058927 |  |  |  | 0.925 |
|  |  | 115,642,633 | rs11102920 |  |  |  | 0.708 |
| 3 | 2 | 6,382,837 | rs12469136 | no gene | 3.10E-06 | 3.81E-006 |  |
|  |  | 6,417,786 | rs898866 |  | 4.44E-06 | 8.94E-006 |  |
|  |  | 6,466,670 | rs7602092 |  | 9.72E-05 | 1.18E-004 |  |
| 4 | 2 | 66,625,504 | rs11897119 | MEIS1 | 4.69E-05 | 1.08E-005 |  |
|  |  | 66,634,957 | rs2300478 |  | 1.61E-12 | 7.77E-016 |  |
|  |  | 66,611,926 | rs6710341* |  |  |  |  |
|  |  | 66,617,812 | rs12469063* |  |  |  |  |
| 5 | 2 | 66,829,004 | rs11678596 | no gene | 1.37E-09 | 2.11E-009 |  |
|  |  | 66,832,460 | rs11683508 |  |  |  | 1 |
|  |  | 66,826,347 | rs7566062 |  |  |  | 0.554 |
| 6 | 2 | 67,923,371 | rs1820987 | no gene | 9.04E-05 | 4.00E-006 |  |
|  |  | 67,923,729 | rs6747972 |  | 2.32E-05 | 1.37E-006 |  |
|  |  | 67,926,267 | rs2116050 |  | 3.08E-05 | 7.84E-006 |  |
| 7 | 2 | 70,992,193 | rs11681519 | VAX2 | 3.75E-05 | 2.94E-005 |  |
|  |  | 71,016,681 | rs17720303 | ATP6V1B1 |  |  | 0.867 |
| 8 | 2 | 75,071,839 | rs7602776 | no gene | 2.77E-05 | 2.14E-005 |  |
|  |  | 75,121,988 | rs2422090 |  |  |  | 0.647 |
|  |  | 75,023,844 | rs11688381 |  |  |  | 0.935 |
| 9 | 2 | 97,739,438 | rs5865 | TMEM131 | 3.25E-05 | 2.05E-005 |  |
|  |  | 97,674,255 | rs1463173 | no gene |  |  | 0.659 |
|  |  | 97,676,314 | rs11123861 | no gene |  |  | 0.653 |
| 10 | 2 | 133,072,868 | rs12618657 | GPR39 | 5.61E-05 | 4.52E-005 |  |
|  |  | 133,084,047 | rs6726461 |  | 1.53E-04 | 1.72E-004 |  |
|  |  | 133,085,386 | rs10496689 |  | 1.53E-04 | 1.72E-004 |  |
| 11 | 6 | 38,473,819 | rs9296249 | BTBD9 | 2.81E-07 | 7.20E-007 |  |
|  |  | 38,473,851 | rs9357271 |  | 2.98E-07 | 6.74E-007 |  |
|  |  | 38,548,948 | rs3923809* |  |  |  |  |
| 12 | 8 | 40,825,954 | rs11778522 | ZMAT4 | 1.57E-06 | 9.57E-006 |  |
|  |  | 40,842,195 | rs16890054 |  |  |  | 0.715 |
|  |  | 40,848,655 | rs6994354 |  | 2.33E-002 | 6.50E-002 | 0.715 |

### Table S 1 continued:

| **Locus** | **Chr** | **Genomic position** | **SNP rs ID** | **Gene** | **MDS**  **λ-corrected P_GWA_** | **VC**  **λ-corrected P_GWA_** | **r^2^** |
| --- | --- | --- | --- | --- | --- | --- | --- |
| 13 | 9 | 955,006 | rs279899 | DMRT1 | 6.40E-06 | 4.80E-005 |  |
|  |  | 965,112 | rs279910 | DMRT3 |  |  | 0.927 |
|  |  | 948,073 | rs10123824 | DMRT1 |  |  | 0.614 |
| 14 | 9 | 4,437,321 | rs10814972 | no gene | 1.39E-04 | 2.07E-004 |  |
|  |  | 4,444,339 | rs10814977 |  | 5.08E-06 | 2.27E-005 |  |
|  |  | 4,445,193 | rs7872012 |  | 5.89E-06 | 1.77E-005 |  |
| 15 | 9 | 8,816,767 | rs10120501 | PTPRD | 5.42E-06 | 8.56E-006 |  |
|  |  | 8,836,955 | rs1975197 |  | 1.13E-04 | 4.94E-005 |  |
|  |  | 9,251,737 | rs4626664* |  |  |  |  |
| 16 | 9 | 24,975,660 | rs1461340 | no gene | 6.59E-05 | 1.24E-005 |  |
|  |  | 24,989,327 | rs1125516 |  | 9.39E-05 | 7.75E-006 |  |
|  |  | 24,989,702 | rs1932418 |  | 7.62E-05 | 7.97E-006 |  |
| 17 | 9 | 128,447,364 | rs11793373 | LMX1B | 4.78E-05 | 8.50E-005 |  |
|  |  | 132,625,367 | rs10793970 | ABL1 | 2.06E-04 | 1.15E-003 |  |
|  |  | 128,419,339 | rs10819189 | LMX1B |  |  | 0.88 |
| 18 | 10 | 4,037,125 | rs4881271 | no gene | 1.25E-04 | 1.36E-003 |  |
|  |  | 4,038,528 | rs7073584 |  | 8.48E-05 | 9.04E-004 |  |
|  |  | 4,040,003 | rs2031577 |  | 1.32E-04 | 1.52E-003 |  |
| 19 | 10 | 54,236,214 | rs7095636 | no gene | 4.34E-05 | 7.75E-005 |  |
|  |  | 54,224,030 | rs4935349 |  |  |  | 0.724 |
|  |  | 54,217,162 | rs12248290 |  |  |  | 0.623 |
| 20 | 12 | 76,800,094 | rs1731754 | NAV3 | 5.56E-05 | 2.22E-005 |  |
|  |  | 76,798,729 | rs1677913 |  |  |  | 0.793 |
|  |  | 76,858,325 | rs1382639 |  |  |  | 0.702 |
| 21 | 12 | 125,063,408 | rs7956291 | no gene | 7.54E-05 | 1.58E-004 |  |
|  |  | 125,039,664 | rs7300194 |  |  |  | 1 |
|  |  | 125,060,256 | rs10847032 |  |  |  | 0.898 |
| 22 | 13 | 85,569,007 | rs7994093 | no gene | 7.20E-05 | 1.32E-004 |  |
|  |  | 85,568,655 | rs1334163 |  |  |  | 0.731 |
|  |  | 85,564,135 | rs17705877 |  |  |  | 0.689 |
| 23 | 15 | 65,823,906 | rs12593813 | MAP2K5 | 1.60E-05 | 1.49E-006 |  |
|  |  | 65,824,632 | rs11635424 | MAP2K5 | 1.65E-05 | 1.52E-006 |  |
|  |  | 65,890,260 | rs6494696* | no gene |  |  |  |
| 24 | 16 | 51,182,239 | rs3104767 | LOC643714 | 7.02E-07 | 7.38E-007 |  |
|  |  | 51,187,203 | rs3104774 |  | 1.31E-06 | 1.36E-006 |  |
|  |  | 51,196,004 | rs3104788 |  | 9.76E-07 | 1.19E-006 |  |

### Table S 1 continued:

| **Locus** | **Chr** | **Genomic position** | **SNP rs ID** | **Gene** | **MDS**  **λ-corrected P_GWA_** | **VC**  **λ-corrected P_GWA_** | **r^2^** |
| --- | --- | --- | --- | --- | --- | --- | --- |
| 25 | 20 | 59,663,684 | rs1006644 | CDH4 | 1.61E-05 | 2.46E-005 |  |
|  |  | 59,655,012 | rs2427223 |  |  |  | 0.778 |
|  |  | 59,669,071 | rs6142824 |  |  |  | 0.736 |
| 26 | 21 | 28,407,477 | rs17747953 | no gene | 1.30E-05 | 1.93E-004 |  |

A star (*) indicates SNPs which had been identified in previous RLS GWAs [2-4]. P-values of the GWA phase are given as λ-corrected nominal P-values. Two different methods for λ correction were applied, multi-dimensional-scaling (MDS)-analysis using PLINK and variance components (VC)-analysis using the EMMAX software with the P values listed in the respective columns “MDS λ-corrected P_GWA_” and “VC λ-corrected P_GWA_”. The selection of SNPs for replication was based on the MDS λ-corrected P values. r^2^-values based on Hapmap CEU data are given for those SNPs which were selected for replication based on their LD with the best-associated SNP in each region. Genomic position and gene annotation refer to the hg18 genome.
