# Supplementary material for: Genome-Wide Association Study Identifies Novel Restless Legs Syndrome Susceptibility Loci on 2p14 and 16q12.1
Source: PLoS Genet. 2011 Jul 14;7(7):e1002171. doi: 10.1371/journal.pgen.1002171 (PMC3136436; doi:10.1371/journal.pgen.1002171)
Supplement: Table S2 — Replication stage association results for individual replication samples. P-values are derived from logistic regression and correcting for gender and age as well as for population stratification where possible (see Materials and Methods). Each replication sample was Bonferroni-corrected using the number of SNPs which passed quality control for the respective sample. The OR refers to the minor allele. NA; SNP could not be analysed due to failing quality control in the respective sample. (DOC) [file pgen.1002171.s007.doc]

### Table S2: Replication stage association results for individual replication samples.

| **Sample** | **GER1** | | |  | **GER2** | | |  | **CZ** | | |  |
| --- | --- | --- | --- | --- | --- | --- | --- | --- | --- | --- | --- | --- |
| **dbSNP ID** | **OR**  **(95% CI)** | **P_nominal_** | **P_lamba-corrected_** | **P**  **_Bonferroni-corrected_** | **OR**  **(95% CI)** | **P_nominal_** | **P_lamba-corrected_** | **P**  **_Bonferroni-corrected_** | **OR**  **(95% CI)** | **P_nominal_** | **P_lamba-corrected_** | **P**  **_Bonferroni-corrected_** |
| rs11897119 | 0.76  (0.67-0.87) | 2.39E-005 | 1.28E-004 | 8.86E-003 | 0.69  (0.6-0.81) | 2.16E-006 | 1.76E-005 | 1.13E-003 | 0.68  (0.53-0.86) | 1.66E-003 | 1.04E-002 | 7.10E-001 |
| rs2300478 | 1.66  (1.46-1.88) | 2.20E-014 | 4.43E-012 | 3.05E-010 | 1.83  (1.56-2.14) | 9.18E-014 | 1.44E-011 | 9.19E-010 | 1.74  (1.33-2.28) | 5.27E-005 | 9.99E-004 | 6.79E-002 |
| rs6747972 | 1.23  (1.1-1.39) | 4.18E-004 | 1.39E-003 | 9.56E-002 | 1.38  (1.19-1.59) | 1.73E-005 | 9.83E-005 | 6.29E-003 | 1.19  (0.94-1.5) | 1.41E-001 | 2.31E-001 | 1 |
| rs2116050 | 1.26  (1.12-1.42) | 8.60E-005 | 3.72E-004 | 2.57E-002 | 1.34  (1.16-1.55) | 6.77E-005 | 3.05E-004 | 1.95E-002 | 1.16  (0.92-1.45) | 2.08E-001 | 3.05E-001 | 1 |
| rs9357271 | 0.75  (0.65-0.87) | 1.03E-004 | 4.30E-004 | 2.96E-002 | 0.59  (0.49-0.71) | 3.47E-008 | 5.75E-007 | 3.68E-005 | 0.67  (0.5-0.89) | 6.39E-003 | 2.64E-002 | 1 |
| rs10120501 | 1.2  (1.07-1.35) | 2.11E-003 | 5.33E-003 | 3.68E-001 | NA | NA | NA | NA | 1.29  (1.03-1.63) | 3.02E-002 | 7.76E-002 | 1 |
| rs1975197 | 1.31  (1.13-1.52) | 3.77E-004 | 1.27E-003 | 8.75E-002 | 1.21  (1.01-1.44) | 3.72E-002 | 5.90E-002 | 1 | 1.29  (0.95-1.75) | 1.02E-001 | 1.84E-001 | 1 |
| rs12593813 | 0.69  (0.61-0.79) | 2.49E-008 | 4.37E-007 | 3.01E-005 | 0.62  (0.53-0.73) | 1.00E-008 | 2.06E-007 | 1.32E-005 | 0.64  (0.5-0.82) | 5.36E-004 | 4.83E-003 | 3.29E-001 |
| rs11635424 | 0.72  (0.63-0.81) | 3.14E-007 | 3.55E-006 | 2.45E-004 | 0.63  (0.54-0.74) | 1.23E-008 | 2.43E-007 | 1.56E-005 | 0.64  (0.5-0.82) | 5.11E-004 | 4.68E-003 | 3.18E-001 |
| rs6494696 | 0.72  (0.63-0.82) | 4.40E-007 | 4.69E-006 | 3.24E-004 | 0.62  (0.53-0.73) | 6.70E-009 | 1.48E-007 | 9.47E-006 | 0.63  (0.49-0.81) | 3.49E-004 | 3.61E-003 | 2.45E-001 |
| rs3104767 | 0.74  (0.65-0.83) | 6.87E-007 | 6.79E-006 | 4.69E-004 | 0.79  (0.68-0.92) | 2.06E-003 | 5.22E-003 | 3.34E-001 | 0.73  (0.57-0.93) | 1.19E-002 | 4.06E-002 | 1 |
| rs3104788 | 0.72  (0.64-0.81) | 1.25E-007 | 1.66E-006 | 1.14E-004 | 0.81  (0.7-0.93) | 4.13E-003 | 9.34E-003 | 5.98E-001 | 0.73  (0.57-0.93) | 1.06E-002 | 3.76E-002 | 1 |

### Table S2 continued:

| **Sample** | **FR** | | | **FIN** | | | **CAN** | | |  | **US** | | |
| --- | --- | --- | --- | --- | --- | --- | --- | --- | --- | --- | --- | --- | --- |
| **dbSNP ID** | **OR**  **(95% CI)** | **P_nominal_** | **P**  **_Bonferroni-corrected_** | **OR**  **(95% CI)** | **P_nominal_** | **P**  **_Bonferroni-corrected_** | **OR**  **(95% CI)** | **P_nominal_** | **P_lamba-corrected_** | **P**  **_Bonferroni-corrected_** | **OR**  **(95% CI)** | **P_nominal_** | **P**  **_Bonferroni-corrected_** |
| rs11897119 | 1.04  (0.81-1.33) | 7.62E-001 | 1 | 0.64  (0.47-0.87) | 5.12E-003 | 3.69E-001 | 0.78  (0.61-1.01) | 5.87E-002 | 1.35E-001 | 1 | NA | NA | NA |
| rs2300478 | 1.35  (1.05-1.74) | 1.79E-002 | 1 | 1.85  (1.32-2.6) | 3.86E-004 | 2.78E-002 | 1.88  (1.42-2.49) | 1.04E-005 | 4.85E-004 | 3.35E-002 | 1.57  (1.31-1.9) | 1.63E-006 | 1.06E-004 |
| rs6747972 | 0.99  (0.78-1.26) | 9.20E-001 | 1 | 0.89  (0.66-1.2) | 4.40E-001 | 1 | 1.31  (1-1.7) | 4.74E-002 | 1.17E-001 | 1 | 1.12  (0.94-1.33) | 1.97E-001 | 1 |
| rs2116050 | 1.01  (0.8-1.28) | 9.41E-001 | 1 | 0.86  (0.64-1.16) | 3.28E-001 | 1 | 1.2  (0.93-1.56) | 1.68E-001 | 2.75E-001 | 1 | 1.13  (0.95-1.33) | 1.75E-001 | 1 |
| rs9357271 | 0.71  (0.53-0.96) | 2.81E-002 | 1 | 0.7  (0.49-0.99) | 4.11E-002 | 1 | 0.68  (0.49-0.94) | 1.86E-002 | 6.25E-002 | 1 | 0.63  (0.51-0.79) | 4.96E-005 | 3.23E-003 |
| rs10120501 | 1.15  (0.9-1.47) | 2.52E-001 | 1 | 1.26  (0.93-1.71) | 1.28E-001 | 1 | 0.87  (0.68-1.11) | 2.63E-001 | 3.76E-001 | 1 | 1.26  (1.06-1.49) | 8.79E-003 | 5.71E-001 |
| rs1975197 | 1.19  (0.89-1.59) | 2.38E-001 | 1 | 1.03  (0.66-1.6) | 9.08E-001 | 6.53E+001 | 1.48  (1.07-2.04) | 1.73E-002 | 5.95E-002 | 1 | 1.28  (1.03-1.57) | 2.37E-002 | 1 |
| rs12593813 | 0.66  (0.51-0.86) | 2.22E-003 | 1.51E-001 | 0.92  (0.65-1.31) | 6.41E-001 | 1 | 0.7  (0.53-0.92) | 1.13E-002 | 4.50E-002 | 1 | 0.81  (0.67-0.97) | 2.13E-002 | 1 |
| rs11635424 | 0.65  (0.5-0.85) | 1.46E-003 | 9.94E-002 | 0.93  (0.66-1.32) | 6.98E-001 | 1 | 0.68  (0.51-0.89) | 6.04E-003 | 2.98E-002 | 1 | 0.85  (0.7-1.02) | 7.34E-002 | 1 |
| rs6494696 | 0.7  (0.53-0.9) | 6.86E-003 | 4.67E-001 | 0.96  (0.67-1.36) | 8.00E-001 | 1 | 0.67  (0.5-0.88) | 4.65E-003 | 2.51E-002 | 1 | 0.85  (0.7-1.02) | 8.27E-002 | 1 |
| rs3104767 | 0.95  (0.75-1.21) | 6.67E-001 | 1 | 0.62  (0.45-0.87) | 6.02E-003 | 4.34E-001 | 0.72  (0.55-0.93) | 1.29E-002 | 4.92E-002 | 1 | 0.69  (0.58-0.83) | 5.86E-005 | 3.81E-003 |
| rs3104788 | 0.95  (0.75-1.21) | 6.67E-001 | 1 | 0.63  (0.45-0.88) | 6.31E-003 | 4.54E-001 | 0.75  (0.57-0.97) | 2.72E-002 | 8.05E-002 | 1 | 0.7  (0.59-0.84) | 9.00E-005 | 5.85E-003 |

P values are derived from logistic regression and correcting for gender and age as well as for population stratification where possible (see Materials and Methods). Each replication sample was Bonferroni-corrected using the number of SNPs which passed quality control for the respective sample. The OR refers to the minor allele. NA; SNP could not be analysed due to failing quality control in the respective sample.
