# Supplementary material for: Genome-Wide Association Study Identifies Novel Restless Legs Syndrome Susceptibility Loci on 2p14 and 16q12.1
Source: PLoS Genet. 2011 Jul 14;7(7):e1002171. doi: 10.1371/journal.pgen.1002171 (PMC3136436; doi:10.1371/journal.pgen.1002171)
Supplement: Table S3 — Power analysis for GWA, replication and joint analysis of GWA and replication. Power calculation was performed using the CaTS power calculator [25] using a prevalence set of 0.08 and an additive genetic model. The significance level α was set at 0.05/74 for replication stage analysis and at 0.05/301,406 for genome-wide significance in the joint analysis of GWA and replication. (DOC) [file pgen.1002171.s008.doc]

### Table S3: Power analysis for GWA, replication and joint analysis of GWA and replication.

| Significance level  α = 6.76 x 10^-4^  (0.05/74 tests) | | MAF | 0.05 to 0.1 | | | 0.15 | | | 0.2 | | | 0.25 | | | 0.3 | | |
| --- | --- | --- | --- | --- | --- | --- | --- | --- | --- | --- | --- | --- | --- | --- | --- | --- | --- |
|  |  | OR | 1.2 | 1.4 | 1.6 | 1.2 | 1.4 | 1.6 | 1.2 | 1.4 | 1.6 | 1.2 | 1.4 | 1.6 | 1.2 | 1.4 | 1.6 |
| Sample | N cases | N controls | Power in % | | | | | | | | | | | | | | |
| GER1 | 1,316 | 1,471 | 3 -9 | 31-72 | 77-99 | 17 | 90 | 100 | 26 | 97 | 100 | 33 | 99 | 100 | 39 | 99 | 100 |
| GER2 | 1,104 | 1,073 | 2-6 | 21-55 | 61-95 | 11 | 78 | 99 | 17 | 89 | 100 | 22 | 94 | 100 | 26 | 96 | 100 |
| FIN | 141 | 360 | 0 | 1-2 | 2-8 | 1 | 4 | 16 | 1 | 7 | 24 | 1 | 9 | 32 | 1 | 11 | 38 |
| CZ | 351 | 597 | 0-1 | 3-11 | 12-38 | 2 | 21 | 61 | 3 | 31 | 76 | 4 | 39 | 84 | 5 | 46 | 89 |
| CA | 285 | 285 | 0-1 | 2-5 | 6-20 | 1 | 10 | 35 | 2 | 15 | 47 | 2 | 19 | 56 | 2 | 23 | 63 |
| US | 556 | 1,200 | 1-3 | 8-29 | 31-75 | 5 | 50 | 93 | 8 | 66 | 98 | 11 | 76 | 99 | 13 | 82 | 100 |
| FR | 182 | 768 | 0-1 | 1-4 | 3-15 | 1 | 8 | 30 | 1 | 13 | 44 | 2 | 18 | 55 | 2 | 22 | 62 |
| Replication combined | 3,935 | 5,754 | 26-65 | 97-100 | 100 | 87 | 100 | 100 | 95 | 100 | 100 | 98 | 100 | 100 | 99 | 100 | 100 |

| Significance level  α = 1.66 x 10^-7^  (0.05/301.406) | | MAF | 0.05 to 0.1 | | | 0.15 | | | 0.2 | | | 0.25 | | | 0.3 | | |
| --- | --- | --- | --- | --- | --- | --- | --- | --- | --- | --- | --- | --- | --- | --- | --- | --- | --- |
|  |  | OR | 1.2 | 1.4 | 1.6 | 1.2 | 1.4 | 1.6 | 1.2 | 1.4 | 1.6 | 1.2 | 1.4 | 1.6 | 1.2 | 1.4 | 1.6 |
| Sample | N cases | N controls | Power in % | | | | | | | | | | | | | | |
| GWA | 954 | 1,814 | 0 | 0-6 | 6-49 | 0 | 19 | 83 | 0 | 36 | 95 | 1 | 51 | 99 | 1 | 62 | 99 |
| GER1 | 1,316 | 1,471 | 0 | 1-10 | 13-65 | 0 | 29 | 92 | 1 | 49 | 98 | 1 | 64 | 100 | 2 | 74 | 100 |
| GER2 | 1,104 | 1,073 | 0 | 0-4 | 6-41 | 0 | 14 | 74 | 0 | 27 | 90 | 0 | 40 | 96 | 1 | 49 | 98 |
| FIN | 141 | 360 | 0 | 0 | 0 | 0 | 0 | 0 | 0 | 0 | 0 | 0 | 0 | 1 | 0 | 0 | 1 |
| CZ | 351 | 597 | 0 | 0 | 0-1 | 0 | 0 | 5 | 0 | 1 | 12 | 0 | 2 | 19 | 0 | 3 | 26 |
| CA | 285 | 285 | 0 | 0 | 0 | 0 | 0 | 1 | 0 | 0 | 3 | 0 | 0 | 5 | 0 | 1 | 7 |
| US | 556 | 1,200 | 0 | 0-1 | 0-10 | 0 | 3 | 32 | 0 | 7 | 54 | 0 | 12 | 69 | 0 | 17 | 79 |
| FR | 182 | 768 | 0 | 0 | 0 | 0 | 0 | 0 | 0 | 0 | 2 | 0 | 0 | 4 | 0 | 0 | 6 |
| GWA + Replication | 4,889 | 7,568 | 2-17 | 77-100 | 100 | 44 | 100 | 100 | 67 | 100 | 100 | 81 | 100 | 100 | 89 | 100 | 100 |

Power calculation was performed using the CaTS power calculator [25] using a prevalence set of 0.08 and an additive genetic model. The significance level α was set at 0.05/74 for replication stage analysis and at 0.05/301,406 for genome-wide significance in the joint analysis of GWA and replication.
