# Supplementary material for: Genome-Wide Association Study Identifies Novel Restless Legs Syndrome Susceptibility Loci on 2p14 and 16q12.1
Source: PLoS Genet. 2011 Jul 14;7(7):e1002171. doi: 10.1371/journal.pgen.1002171 (PMC3136436; doi:10.1371/journal.pgen.1002171)
Supplement: Table S4 — Results of TOX3 and BC034767 mutation screening. * “A” refers to the mutant allele, “B” to the reference allele. Position refers to hg18 genome annotation. Codon numbering refers to the reference sequence NM_001146188. Data of the 1000 genomes project was obtained from the November 2010 release via the 1000 genomes browser (http://browser.1000genomes.org/index.html). (DOC) [file pgen.1002171.s009.doc]

**Table S4: Results of *TOX3* and *BC034767* mutation screening.**

| Region | Position on chr 16 | Triplett and amino acid exchange | Codon | Genotype Frequency* | | 1000 genomes |
| --- | --- | --- | --- | --- | --- | --- |
|  |  |  |  | Cases | Controls |  |
|  |  |  |  | AA/AB/BB | AA/AB/BB |  |
| *TOX3* exon 4 | 51,041,929 | (C/T)GG – Arg **>** Trp | 147 | 0/1/722 | 0/0/720 | NA |
| *TOX3* exon 4 | 51,041,885 | AC(C/T) – Thr **=** Thr | 161 | 0/14/709 | 0/11/708 | NA |
| *TOX3* exon 4 | 51,041,787 | A(G/A)T - Ser **>** Asn | 193 | 0/1/724 | 0/0/723 | NA |
| *TOX3* exon 5 | 51,037,614 | GC(C/T) – Ala **=** Ala | 233 | 0/1/716 | 0/0/715 | NA |
| *TOX3* exon 7 | 51,031,316 | AA(C/T) – Asn **=** Asn | 351 | 0/1/723 | 0/0/722 | NA |
| *TOX3* exon 7 | 51,031,085 | CC(C/T) – Pro **=** Pro | 428 | 0/15/709 | 0/15/707 | NA |
| *TOX3* exon 7 | 51,030,950 | AT(C/A) – Ile **=** Ile | 473 | 0/15/707 | 0/7/715 | low_coverage:CEU:status |
| *TOX3* exon 7 | 51,030,832 | (C/T)GC – Arg **>** Cys | 513 | 0/9/706 | 0/10/708 | NA |
| *TOX3* exon 8 | 51,030,274 | intronic | NA | 0/1/717 | 0/0/727 | NA |
| *BC034767* exon 1 | 51,198,344 | C/T | NA | 0/1/724 | 0/0/722 | NA |
| *BC034767* exon 1 | 51,198,021 | C/T | NA | 0/7/718 | 0/4/717 | NA |
| *BC034767* exon 2 | 51,182,470 | C/T | NA | 0/2/724 | 0/1/721 | NA |

* “A” refers to the mutant allele, “B” to the reference allele. Position refers to hg18 genome annotation. Codon numbering refers to the reference sequence NM_001146188. Data of the 1000 genomes project was obtained from the November 2010 release via the 1000 genomes browser (http://browser.1000genomes.org/index.html).
