# Supplementary material for: Genome-Wide Association Study Identifies Novel Restless Legs Syndrome Susceptibility Loci on 2p14 and 16q12.1
Source: PLoS Genet. 2011 Jul 14;7(7):e1002171. doi: 10.1371/journal.pgen.1002171 (PMC3136436; doi:10.1371/journal.pgen.1002171)
Supplement: Table S5 — Prediction of genetic risk; training- and test-set approach. Inclusion threshold P-values were derived from a logistic regression with age and sex as covariates in the training sample. # SNPs indicates the number of SNPs passing the inclusion threshold. Based on these association results, the sum score of SNPs showing the most significant effects (i.e. the number of risk alleles over all SNPs) weighted by the ln(OR) of these effects was chosen as predictor variable in the test set. Based on this sum score, an AUC and Nagelkerke's R were calculated. (DOC) [file pgen.1002171.s010.doc]

### Table S5: Prediction of genetic risk; training and test set approach.

| P-value threshold  P<= | # SNPs | P-value for risk score | Nagelkerke's R² | AUC in % |
| --- | --- | --- | --- | --- |
| 1.00E+000 | 76352 | 1.04E-021 | 0.0659 | 63.80 |
| 9.00E-001 | 68914 | 9.50E-022 | 0.0660 | 63.80 |
| 8.00E-001 | 61427 | 1.20E-021 | 0.0656 | 63.77 |
| 7.00E-001 | 54000 | 3.24E-021 | 0.0641 | 63.65 |
| 6.00E-001 | 46618 | 7.14E-022 | 0.0664 | 63.92 |
| 5.00E-001 | 39318 | 3.27E-021 | 0.0641 | 63.71 |
| 4.00E-001 | 31707 | 1.24E-020 | 0.0622 | 63.51 |
| 3.00E-001 | 24258 | 1.61E-018 | 0.0549 | 62.74 |
| 2.00E-001 | 16528 | 7.22E-017 | 0.0493 | 61.91 |
| 1.00E-001 | 8653 | 2.06E-015 | 0.0442 | 61.46 |
| 5.00E-002 | 4500 | 4.32E-013 | 0.0366 | 60.56 |
| 1.00E-002 | 996 | 1.39E-005 | 0.0131 | 56.26 |
| 1.00E-003 | 126 | 1.28E-002 | 0.0042 | 53.45 |
| 1.00E-004 | 19 | 9.33E-001 | 4.78E-006 | 49.83 |
| 1.00E-005 | 1 | 7.50E-001 | 7.14E-005 | 50.65 |

Inclusion threshold P-values were derived from a logistic regression with age and sex as covariates in the training sample. # SNPs indicates the number of SNPs passing the inclusion threshold. Based on these association results, the sum score of SNPs showing the most significant effects (i.e. the number of risk alleles over all SNPs) weighted by the ln(OR) of these effects was chosen as predictor variable in the test set. Based on this sum score, an AUC and Nagelkerke’s R were calculated.
