# Supplementary material for: Genome-Wide Association Study Identifies Novel Restless Legs Syndrome Susceptibility Loci on 2p14 and 16q12.1
Source: PLoS Genet. 2011 Jul 14;7(7):e1002171. doi: 10.1371/journal.pgen.1002171 (PMC3136436; doi:10.1371/journal.pgen.1002171)
Supplement: Table S6 — Demographic data of GWA and replication samples. Mean age, mean age of onset and respective standard deviations and ranges are given in years. N: number of individuals; SD: standard deviation; AAO: age of onset. GWA: Genome-wide association study; CZ: Czechia; FR: France; FIN: Finland; CA: Canada; US: United States. - indicates that this information is not applicable for the respective sample. (DOC) [file pgen.1002171.s011.doc]

### Table S6: Demographic data of GWA and replication samples.

| **Study phase** | **GWA** | | **Replication** | | | | | |
| --- | --- | --- | --- | --- | --- | --- | --- | --- |
| Sample | cases | controls | GER1  cases | GER1 controls | GER2  cases | GER2 controls | CZ  cases | CZ  controls |
| Total N (% female) | 954 (73.6) | 1814 (51.3) | 1316 (68.3) | 1471 (52.2) | 1104 (70.5) | 1073 (53.4) | 351 (61.8) | 597 (64.0) |
| Mean age (SD) | 60.4 (± 12.5) | 60.9 (± 8.9) | 60.6 (± 13.0) | 49.9 (± 14.5) | 65.3 (±10.5) | 52.9 (±13.6) | 56.6 (± 14.9) | 45.7 (± 7.9) |
| Age range | 6-92 | 33-81 | 7-87 | 32-81 | 29-90 | 26-74 | 12-91 | 18-61 |
| Mean AAO of RLS | 30.3 (± 20.4) | - | 42.7 (± 17.3) | - | 57.3 | - | 39.2 (± 18.2) | - |
| AAO range | 4-85 | - | 3-84 | - | 21-85 | - | 5-82 | - |
| N unknown age | 0 | 12 | 1 | 0 | 2 | 4 | 4 | 0 |
| N unknown AAO of RLS | 159 | - | 257 | - | 10 | - | 29 | - |
| Family history of RLS  (N positive/N negative/N unknown) | 779/77/98 | - | 448/688/180 | - | 315/737/52 | - | 131/214/6 | - |

| **Study phase** | **Replication** | | | | | | | |
| --- | --- | --- | --- | --- | --- | --- | --- | --- |
| Sample | FR  cases | FR  controls | FIN  cases | FIN  controls | CA cases | CA controls | US  cases | US controls |
| Total N (% female) | 182 (60.4) | 768 (50.0) | 141 (73.8) | 360 (57.8) | 285 (62.5) | 285 (60.0) | 556 (61.3) | 1200 (44.7) |
| Mean age (SD) | 55.1 (± 13.7) | 62.4 (± 10.1) | 48.6 (± 16.7) | 46.4 (± 11.9) | 53.6 (± 11.7) | 48.2 (± 17.2) | 63.3 (±13.5) | 52.1 (± 8.0) |
| Age range | 10-91 | 38-90 | 5-82 | 25-73 | 22-93 | 19-89 | 17-93 | 33-75 |
| AAO of RLS | 39.1 (± 16.4) | - | 23.3 (± 14.9 | - | 29.9 (± 16.3) | - | 40.0 (± 20.0) | - |
| AAO range | 6-85 | - | 2-70 | - | 1-79 | - | 2-86 | - |
| N unknown age | 0 | 15 | 0 | 2 | 0 | 13 | 3 | 22 |
| N unknown AAO of RLS | 36 | - | 6 | - | 61 | - | 56 | - |
| Family history of RLS  (N positive/N negative/N unknown) | 64/85/33 | - | 110/22/9 | - | 182/52/51 | - | 363/162/31 | - |

Mean age, mean age of onset and respective standard deviations and ranges are given in years. N: number of individuals; SD: standard deviation; AAO: age of onset. GWA: Genome-wide association study; CZ: Czechia; FR: France; FIN: Finland; CA: Canada; US: United States.

- indicates that this information is not applicable for the respective sample.
